# Rising burden of pancreatic cancer in China: Trends, drivers, and future projections

**DOI:** 10.1371/journal.pone.0327009

**Published:** 2025-07-01

**Authors:** Zhouwei Zhan, Xiuhui Zheng, Shaohua Xu, Hanchen Zheng, Lina Zheng, Jie Wang, Hui Lin, Jiami Yu, Zengqing Guo, Bijuan Chen

**Affiliations:** 1 Department of Medical Oncology, Clinical Oncology School of Fujian Medical University, Fujian Cancer Hospital, Fuzhou, Fujian, China; 2 Department of Medical Affairs, Clinical Oncology School of Fujian Medical University, Fujian Cancer Hospital, Fuzhou, Fujian, China; 3 Department of Hepatobiliary and Pancreatic Surgery, Clinical Oncology School of Fujian Medical University, Fujian Cancer Hospital, Fuzhou, Fujian, China; 4 Department of Radiation Oncology, Clinical Oncology School of Fujian Medical University, Fujian Cancer Hospital, Fuzhou, Fujian, China; National Center for Chronic and Noncommunicable Disease Control and Prevention, Chinese Center for Disease Control and Prevention, CHINA

## Abstract

Pancreatic cancer is one of the most lethal malignancies globally, with increasing incidence and mortality trends. In China, the disease burden has escalated over the past three decades, yet comprehensive national assessments remain limited. This study aims to evaluate the long-term trends, driving factors, and future projections of pancreatic cancer burden in China from 1990 to 2021. Data on incidence, prevalence, mortality, disability-adjusted life years (DALYs), years lived with disability (YLDs), and years of life lost (YLLs) were extracted from the Global Burden of Disease (GBD) Study 2021. Temporal trends were analyzed using Joinpoint regression and age-period-cohort models, while decomposition analysis quantified the contributions of population aging, growth, and epidemiological change. Bayesian age-period-cohort (BAPC) models were applied to project disease burden trends through 2030. In 2021, pancreatic cancer accounted for 118,665 new cases and over 2.9 million DALYs in China, with a significantly higher burden among males. Age-standardized rates of incidence, prevalence, and DALYs increased markedly between 1990 and 2021, outpacing global trends. The burden was concentrated in older age groups and driven primarily by years of life lost. Joinpoint regression identified periods of accelerated increase after 2015. Age-period-cohort analysis revealed that disease burden rises sharply after age 50 and is highest among more recent birth cohorts. Decomposition analysis showed that population aging and epidemiological transitions were the main contributors to increased burden. Projections using BAPC models indicate that incidence, prevalence, and DALY rates will continue to rise through 2030. The burden of pancreatic cancer in China has increased substantially over the past three decades and is projected to rise further. These findings highlight the need for intensified public health interventions focused on prevention, early detection, and effective treatment strategies.

## Introduction

Pancreatic cancer is recognized globally as a malignancy with one of the highest case-fatality ratios, largely due to its insidious onset, late clinical presentation, and limited therapeutic options [1]. Despite accounting for only a small fraction of overall cancer incidence, it ranks among the leading causes of cancer-related mortality worldwide [1]. In recent years, improvements in the diagnosis and management of many solid tumors have not been paralleled in pancreatic cancer, resulting in stagnant survival rates. Globally, the age-standardized mortality rate of pancreatic cancer remains high, and projections suggest a continuous increase in its incidence and disease burden, especially in countries undergoing rapid demographic and epidemiologic transitions [2,3]. This trend is particularly alarming in middle-income countries, where public health infrastructure often lags behind the pace of disease emergence, limiting the potential for early detection and timely intervention. Given these global dynamics, detailed national-level assessments are essential to tailor responses based on regional patterns and needs.

China, as the world’s most populous nation, has witnessed significant shifts in its cancer landscape over the past three decades. Alongside urbanization, aging, and lifestyle changes such as dietary transition and smoking, the burden of pancreatic cancer has increased steadily [4]. Previous analyses have shown substantial rises in cancer mortality in China, with pancreatic cancer emerging as a notable contributor [5]. However, few studies have comprehensively evaluated long-term temporal trends and the complex drivers underlying these shifts using high-quality, nationally representative data. The Global Burden of Disease (GBD) Study offers a unique opportunity to conduct such assessments, providing consistent, comparative, and methodologically harmonized estimates of cancer burden over time and across regions [6,7]. Utilizing GBD 2021 data, this study aims to explore the evolving patterns of pancreatic cancer burden in China, identify contributing factors through decomposition and regression modeling, and project future trends using advanced epidemiologic forecasting.

Understanding the epidemiological trajectory of pancreatic cancer is critical for guiding public health strategies and optimizing resource allocation. Given its aggressive nature and poor prognosis, the increasing burden of pancreatic cancer poses significant clinical and societal challenges. Unlike cancers amenable to screening, pancreatic cancer lacks reliable early detection methods, and therapeutic advancements have offered limited survival gains [8]. Consequently, much of the disease burden is attributed to premature mortality, as reflected in the high proportion of years of life lost (YLLs) relative to years lived with disability (YLDs) in disability-adjusted life years (DALYs) metrics. Age-period-cohort and Joinpoint regression analyses can offer nuanced insights into risk trajectories by disentangling the effects of demographic changes and generational risk exposure. Additionally, Bayesian modeling approaches such as BAPC enable dynamic forecasting that can inform anticipatory policy measures. By combining these methods, this study provides a comprehensive evaluation of the past, present, and projected future of pancreatic cancer in China. The findings will offer critical evidence for decision-makers to prioritize early intervention, promote modifiable risk factor control, and guide investment in diagnostic and therapeutic infrastructure tailored to the rising pancreatic cancer challenge.

## Methods

### Data sources

Data for this study were obtained exclusively from the GBD Study 2021, an ongoing multinational collaborative effort coordinated by the Institute for Health Metrics and Evaluation (IHME). The GBD provides comprehensive estimates of the incidence, prevalence, mortality, and DALYs for 371 diseases and injuries across 204 countries and territories, disaggregated by age, sex, year, and location [6,7,9]. No additional external datasets were incorporated. For this study, we extracted data specific to pancreatic cancer in China from 1990 to 2021, including age-standardized and all-age estimates of incidence, prevalence, deaths, DALYs, YLDs, and YLLs. All estimates were produced using a standardized and validated methodological framework developed by IHME. This framework integrates multiple data sources, including vital registration systems, cancer registries, verbal autopsy reports, and household surveys, and applies statistical modeling to ensure consistency across locations and years. Cause-specific mortality estimates were generated using the Cause of Death Ensemble model (CODEm), which synthesizes multiple models and covariates to enhance predictive accuracy. Non-fatal outcomes, including incidence, prevalence, and YLDs, were estimated using DisMod-MR 2.1, a Bayesian meta-regression tool that ensures internal consistency across disease parameters. These models incorporate a range of covariates, such as smoking prevalence, alcohol use, body mass index, and diabetes, to improve precision. However, as our study focused on the descriptive analysis of disease burden rather than causal relationships, identification and adjustment for confounding factors were not applicable. Uncertainty intervals (UIs) were calculated based on 1,000 posterior draws to reflect model uncertainty [6,7,10]. Age-standardized rates were computed using the GBD global standard population, enabling valid comparisons across years without being influenced by demographic shifts. All data used were accessed from the Global Health Data Exchange (GHDx), ensuring transparency and reproducibility of the analysis.

### Definition and estimation

Pancreatic cancer was defined according to the International Classification of Diseases, 10th Revision (ICD-10), using the code C25, which includes malignant neoplasms of the pancreas [11]. The GBD Study 2021 estimates the burden of pancreatic cancer through a standardized framework incorporating both fatal and non-fatal outcomes. For fatal estimates, mortality data were modeled using the CODEm, which selects optimal combinations of predictive covariates and model structures to estimate cause-specific mortality while accounting for data quality, completeness, and regional discrepancies. Non-fatal estimates, including incidence, prevalence, and YLDs, were modeled using the DisMod-MR 2.1 Bayesian meta-regression tool, which ensures internal consistency among epidemiological parameters and propagates uncertainty across all stages of estimation. DALYs were calculated as the sum of YLLs due to premature death and YLDs, capturing both fatal and non-fatal health loss. YLLs were derived by multiplying the number of deaths by the standard life expectancy at the age of death, based on the GBD standard life table. YLDs were estimated by multiplying the prevalence of pancreatic cancer by a fixed disability weight assigned to a single sequela that represents the average health state of individuals living with pancreatic cancer. This sequela includes the overall morbidity associated with the disease, such as functional limitations, symptoms, and treatment-related impacts. The disability weight was derived from population-based surveys conducted as part of the GBD study, reflecting the severity of health loss as perceived by the general public. Age-standardized rates for incidence, prevalence, mortality, DALYs, YLDs, and YLLs were calculated using the GBD global standard population to allow for cross-time and cross-country comparability, minimizing bias from changes in demographic structure. Robustness of the GBD estimates was ensured through internal validation methods built into the CODEm and DisMod-MR 2.1 models, including cross-validation, predictive performance checks, and covariate sensitivity testing. UIs were generated based on 1,000 posterior draws and are reported for all estimates to reflect model variability.

### Descriptive analysis

A descriptive analysis was performed to assess the burden of pancreatic cancer in China from 1990 to 2021. Metrics evaluated included incidence, prevalence, mortality, YLDs, YLLs, and DALYs. Both all-age counts and age-standardized rates (ASRs) per 100,000 population were analyzed. Age standardization was carried out using the GBD global reference population to ensure comparability over time by adjusting for demographic shifts. The data were stratified by sex and grouped into 5-year age intervals to examine variations across demographic subgroups. Age-specific case numbers and crude rates were compiled for the years 1990 and 2021 to describe changes in disease burden across age groups. Trends in age-standardized incidence, prevalence, and mortality rates were plotted to illustrate changes over the study period, with sex-specific differences highlighted. Absolute and percentage changes in ASRs were calculated to evaluate the relative burden in China compared to global averages. Additionally, the age and sex distribution of burden in 2021 was illustrated to identify peak burden age groups and sex disparities across indicators. These findings were interpreted in the context of demographic changes, particularly population aging and sex-specific exposure to risk factors. All descriptive analyses were conducted using publicly available estimates from the GBD 2021 database, and data visualization was completed using R software (version 4.3.1).

### Joinpoint regression analysis

Joinpoint regression analysis was employed to evaluate temporal trends in the age-standardized incidence, prevalence, and mortality rates of pancreatic cancer in China from 1990 to 2021. This method was used to detect points in time at which significant changes in trend occurred and to quantify the rate of change within each identified segment. The Joinpoint Regression Program (version 5.2.0; National Cancer Institute, USA) was used to perform the analysis [12–14]. Annual percent changes (APCs) and average annual percent changes (AAPCs) were calculated for each metric. APCs were estimated for individual trend segments between joinpoints, while AAPCs represented the average rate of change over the entire study period. A Monte Carlo permutation method was applied to determine the number and location of joinpoints, with a maximum of five joinpoints allowed. Statistically significant changes were identified when the two-sided *p*-value was less than 0.05, and 95% confidence intervals (CIs) were used to assess the precision of the estimates. Separate analyses were conducted for males, females, and the total population to examine sex-specific trends. Age-standardized rates obtained from the GBD 2021 database were used as input for all joinpoint models. Results were used to identify periods of trend acceleration or deceleration and to support the interpretation of shifts in disease burden over time, including periods of stagnation or resurgence, as observed in the descriptive analysis.

### Age-period-cohort (APC) analysis

An APC analysis was conducted to disentangle the independent effects of age, time period, and birth cohort on the temporal trends of pancreatic cancer in China. This modeling approach was used to assess how changes in incidence, prevalence, and DALY rates could be attributed to aging, period-related influences (such as medical advancements or policy changes), and cohort-specific exposures or behaviors over time [15]. Age-specific, period-specific, and cohort-specific data were extracted from the GBD 2021 database. The analysis was carried out using a log-linear Poisson model framework, with data stratified into 5-year age groups (15–19 to 90–94 years) and 5-year time intervals (1992–2021). Birth cohorts were derived by subtracting the midpoint of each age group from the midpoint of each period. To address the identifiability problem arising from the linear dependency among age, period, and cohort, the intrinsic estimator (IE) method was applied. This method has been shown to provide unbiased and robust estimates of independent age, period, and cohort effects and has been widely used in epidemiological research [16–19]. The APC analysis was performed separately for incidence, prevalence, and DALY rates, and stratified by sex to explore gender-specific trends. The results were interpreted to identify patterns such as age groups with elevated risks, historical periods associated with increases or decreases in burden, and generational shifts that may reflect long-term exposures. All analyses were conducted using R software (version 4.3.1), with the “Epi” and “apc” packages utilized for modeling.

### Decomposition analysis

A decomposition analysis was conducted to quantify the relative contributions of three primary drivers (population aging, population growth, and epidemiological changes) to the observed increases in the incidence, prevalence, and DALY rates of pancreatic cancer in China between 1990 and 2021. This method was used to disaggregate the net change in disease burden into additive components attributable to demographic and epidemiologic dynamics [20,21]. For this analysis, changes in the number of incident cases, prevalent cases, and DALYs were decomposed into contributions from: (1) population growth, which reflects the increase in the total number of individuals; (2) population aging, which captures shifts in age distribution toward older groups with higher cancer risk; and (3) changes in age-specific rates, defined as epidemiological changes, representing shifts in the underlying disease risk or health system performance independent of demographic structure. The decomposition was performed using a stepwise replacement algorithm, where the effect of each factor was calculated by sequentially substituting the population size, age structure, and age-specific rates of 1990 with those of 2021. Analyses were conducted separately by sex to examine gender-specific contributions. This approach enabled a clearer understanding of whether the rising burden was driven predominantly by aging demographics, expanding population size, or changing disease risk profiles.

### Bayesian age-period-cohort (BAPC) analysis

A BAPC analysis was performed to project the future burden of pancreatic cancer in China through 2030. This approach was selected for its ability to incorporate historical trends and uncertainty in a coherent probabilistic framework, enabling the estimation of future age-standardized rates for incidence, prevalence, and DALYs based on prior observations. Data on age-specific rates from 1990 to 2021 were extracted from the GBD 2021 database and structured into consecutive 5-year age groups and 5-year calendar periods. The BAPC model was fitted using integrated nested Laplace approximations (INLA), which allow for efficient and accurate Bayesian inference. Independent random walk priors of second order were assigned to the age, period, and cohort effects to reflect the smooth temporal evolution typically observed in epidemiological data [22,23]. The model was implemented using the BAPC and INLA packages in R software (version 4.3.1). Sex-specific projections were generated, and results were reported as median posterior estimates with corresponding 95% credible intervals. This analysis provided evidence-based estimates of future pancreatic cancer trends under current trajectories, supporting policy formulation and strategic planning for disease prevention and control.

### Ethics approval

This study utilized publicly available, de-identified GBD 2021 data, eliminating the need for ethical approval or consent.

## Results

### Overview of pancreatic cancer burden in China, 2021

In 2021, pancreatic cancer continued to pose a significant public health burden in China, with both incidence and mortality closely aligned, reflecting the disease’s high lethality. A total of approximately 118,665 new cases were reported, with a higher burden observed in males than females. The age-standardized incidence rate was 5.64 per 100,000 population, with men experiencing a substantially higher rate (7.29) compared to women (4.18). Similarly, the age-standardized prevalence rate was 4.53 per 100,000, again disproportionately higher in males. Mortality mirrored the incidence figures, with 119,602 deaths and an age-standardized death rate of 5.72 per 100,000, further highlighting the poor prognosis associated with this malignancy. The burden measured by DALYs reached over 2.9 million, with men bearing nearly two-thirds of this burden. Age-standardized DALY rates stood at 137.23 per 100,000, driven primarily by YLLs, which far outweighed YLDs, underscoring the disease’s rapid progression and fatal outcomes. These statistics emphasize the urgent need for early detection and effective interventions for pancreatic cancer in China (Table 1).

**Table 1:** All-age cases and age-standardized incidence, prevalence, deaths, DALYs, YLDs, and YLLs rates in 2021 for pancreatic cancer in China.

Measure	All-ages cases			Age-standardized rates per 100,000 people		
	Total	Male	Female	Total	Male	Female
Incidence	118665 (94623, 144663)	72280 (54334, 92975)	46386 (34923, 59339)	5.64 (4.52, 6.84)	7.29 (5.55, 9.24)	4.18 (3.15, 5.34)
Prevalence	95524 (75563, 116662)	59943 (44928, 77312)	35581 (26783, 45781)	4.53 (3.6, 5.5)	5.92 (4.5, 7.54)	3.22 (2.43, 4.14)
Deaths	119602 (95654, 145218)	72159 (54384, 92255)	47443 (35778, 60492)	5.72 (4.59, 6.91)	7.37 (5.64, 9.3)	4.29 (3.23, 5.46)
DALYs	2930317 (2301049, 3575079)	1854033 (1382248, 2393909)	1076284 (806636, 1392992)	137.23 (108.15, 166.74)	179.36 (134.98, 229.1)	96.89 (72.71, 125.18)
YLDs	24697 (15891, 34348)	15092 (9603, 21694)	9604 (5859, 14037)	1.17 (0.75, 1.61)	1.5 (0.95, 2.14)	0.86 (0.53, 1.26)
YLLs	2905620 (2284585, 3543741)	1838941 (1371658, 2372661)	1066679 (800943, 1379845)	136.06 (107.36, 165.26)	177.86 (133.91, 227.18)	96.03 (72.16, 124)

Values in parentheses indicate 95% UIs, estimated using Monte Carlo simulations. Abbreviations: DALYs, disability-adjusted life-years; YLDs, years lived with disability; YLLs, years of life lost.

### Age and sex distribution of pancreatic cancer in China, 2021

**Fig 1:**
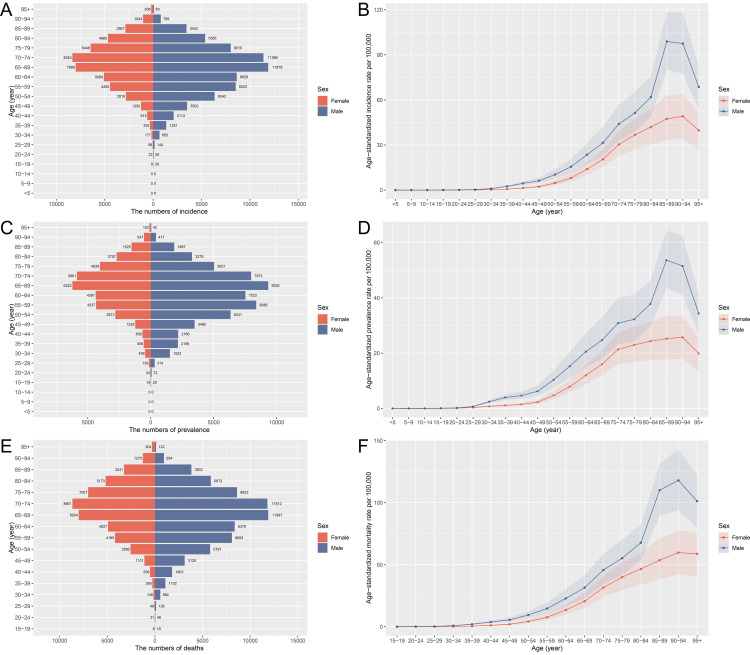
Age and sex distribution of deaths, prevalence, and incidence rates of pancreatic cancer in China in 2021. (A) Age-specific and gender-specific number of incident cases of pancreatic cancer. (B) Age-specific and gender-specific rate of incidence of pancreatic cancer per 100,000 people. (C) Age-specific and gender-specific number of prevalent cases of pancreatic cancer. (D) Age-specific and gender-specific rate of prevalence of pancreatic cancer per 100,000 people. (E) Age-specific and gender-specific number of pancreatic cancer deaths. (F) Age-specific and gender-specific rate of deaths due to pancreatic cancer per 100,000 people.

The age and sex distribution of pancreatic cancer incidence, prevalence, and mortality in 2021 showed distinct patterns. The total number of cases and deaths increased with age, peaking in the 65–69 age group for both sexes. However, age-specific rates of incidence, prevalence, and mortality per 100,000 people peaked in much older age groups, notably among those aged 85–94, with a clear predominance in males across all age groups. Prevalence data mirrored these trends, with case numbers highest in the 65–69 age group but rates peaking among the oldest age groups, reflecting both the increasing incidence with age and greater male burden across age categories (Fig 1). The total DALYs due to pancreatic cancer were substantial, driven primarily by YLLs, reflecting the high mortality associated with this cancer. YLLs were concentrated in the 65–69 age group, with rate peaks in the oldest age categories (85–94 years), where males consistently showed higher values. Although the non-fatal burden (YLDs) was relatively low, it too peaked in the 65–69 age group, indicating an increase in disease duration or severity in later life stages. The gender disparity remained evident, with males bearing a higher burden in all age-related measures of DALYs, YLDs, and YLLs (S1 Fig).

### Temporal trends in pancreatic cancer burden in China, 1990–2021

**Fig 2:**
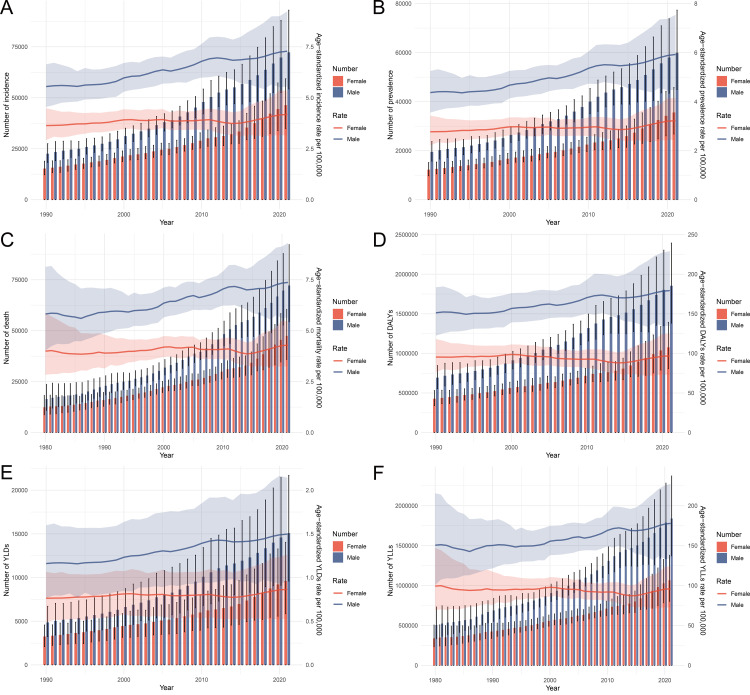
Temporal trends in the incidence, prevalence, deaths, DALYs, YLDs, and YLLs of pancreatic cancer in China from 1990 to 2021, by sex. (A) The number of incident cases and age-standardized incidence rates per 100,000 people from 1990 to 2021. (B) The number of prevalent cases and age-standardized prevalence rates per 100,000 people from 1990 to 2021. (C) The number of deaths and age-standardized mortality rates per 100,000 people from 1990 to 2021. (D) The number of DALYs and age-standardized DALY rates per 100,000 people from 1990 to 2021. (E) The number of YLDs and age-standardized YLD rates per 100,000 people from 1990 to 2021. (F) The number of YLLs and age-standardized YLL rates per 100,000 people from 1990 to 2021. Abbreviations: DALYs, disability-adjusted life years; YLDs, years lived with disability; YLLs, years of life lost.

The temporal trends in pancreatic cancer burden from 1990 to 2021 in China reveal a notable increase across incidence, prevalence, and mortality metrics. Both the number of new cases and age-standardized incidence rates per 100,000 people showed a consistent upward trend, with males experiencing higher values than females throughout the period (Fig 2A). Similarly, the prevalence of pancreatic cancer, measured both by the number of cases and age-standardized prevalence rates, increased over time, with male rates consistently exceeding female rates (Fig 2B). Mortality trends reflected similar patterns, with both the number of deaths and age-standardized death rates showing a marked increase over the study period, again with males experiencing a higher burden (Fig 2C). The disease burden, quantified through DALYs, YLDs, and YLLs, has escalated substantially from 1990 to 2021. The DALYs and age-standardized DALY rates per 100,000 people have shown pronounced increases, with a higher burden observed in males (Fig 2D). Non-fatal outcomes, represented by YLDs, also followed an upward trend, with slightly higher values in males (Fig 2E). Fatal outcomes, as indicated by YLLs, contributed the most significantly to the overall burden, showing substantial increases over time, with males bearing a greater impact than females (Fig 2F).

### Age distribution of pancreatic cancer burden in China, 1990 and 2021

The age distribution of pancreatic cancer burden in 1990 and 2021 highlights shifts in the disease’s impact across different age groups over time. For incidence, the number of new cases and crude rates per 100,000 people were notably higher in 2021 than in 1990, particularly in older age groups, with the highest rates observed among those aged 85–94 (S2A Fig). Mortality and prevalence patterns mirrored these trends, showing an increase in both numbers and crude rates with advancing age, with substantial rises in the oldest age groups from 1990 to 2021 (S2B and S2C Figs). The burden of DALYs, reflecting both premature mortality and disability, was also highest in older age groups, with a more pronounced increase observed in 2021, particularly among those over 65 years (S2D Fig). S2E and S2F Figs provide additional insights into the non-fatal and fatal burdens of pancreatic cancer over this period. The YLDs, which represent the non-fatal burden, were highest in the older age groups, with males consistently showing higher values than females. The YLLs, representing the fatal component of disease burden, increased markedly with age, particularly among males, indicating a significant impact of premature mortality in the older population. These trends collectively demonstrate the intensifying burden of pancreatic cancer over time, especially among older males, and underscore the disease’s growing impact on public health in China.

### Trends in pancreatic cancer burden in China and globally, 1990–2021

Between 1990 and 2021, the age-standardized burden of pancreatic cancer in China exhibited a marked upward trajectory, significantly outpacing global averages. As shown in Table 2, China experienced notable increases in the age-standardized rates of incidence, prevalence, and mortality, with the incidence rate rising from 4.54 to 5.64 per 100,000 and mortality increasing from 4.83 to 5.72 per 100,000. These trends were accompanied by a substantial rise in DALYs, from 123.16 to 137.23 per 100,000, primarily driven by increases in YLLs, which consistently dominated over YLDs. In contrast, global rates showed only modest changes or remained nearly stable over the same period, with DALYs essentially plateauing. These disparities are clearly visualized in S3 Fig, where China’s burden curves for age-standardized incidence, mortality, and DALYs all show steeper upward trends compared to the relatively flatter global curves. This divergence suggests that China’s escalating burden may be influenced by rapid demographic shifts, lifestyle transitions, and limited early detection, emphasizing the urgency for enhanced public health strategies tailored to pancreatic cancer prevention and management in China.

**Table 2:** Change of age-standardized rates in incidence, prevalence, deaths, DALYs, YLDs, and YLLs for pancreatic cancer between 1990 and 2021 in China and global level.

Measure	China			Global		
	1990	2021	Change	1990	2021	Change
Incidence	4.54 (3.84, 5.29)	5.64 (4.52, 6.84)	0.72 (0.50 - 0.94)^*^	5.47 (5.16, 5.73)	5.96 (5.39, 6.42)	0.28 (0.17 - 0.39)^*^
Prevalence	3.55 (2.99, 4.14)	4.53 (3.6, 5.5)	0.80 (0.64 - 0.96)^*^	4.39 (4.15, 4.6)	5.12 (4.66, 5.5)	0.49 (0.37 - 0.61)^*^
Deaths	4.83 (4.1, 5.61)	5.72 (4.59, 6.91)	0.49 (0.29 - 0.69)^*^	5.66 (5.33, 5.93)	5.95 (5.4, 6.41)	0.20 (0.11 - 0.28)^*^
DALYs	123.16 (103.69, 143.27)	137.23 (108.15, 166.74)	0.36 (0.18 - 0.54)^*^	129.32 (122.98, 135.98)	130.33 (120.52, 140.13)	0.02 (−0.10 - 0.15)
YLDs	0.95 (0.63, 1.3)	1.17 (0.75, 1.61)	0.66 (0.47 - 0.86)^*^	1.11 (0.78, 1.43)	1.21 (0.85, 1.6)	0.28 (0.19 - 0.36)^*^
YLLs	122.2 (102.85, 142.16)	136.06 (107.36, 165.26)	0.29 (0.08 - 0.50)^*^	128.22 (121.98, 134.81)	129.12 (119.47, 138.77)	−1.31 (−1.36 - −1.27)^*^

Values in parentheses indicate 95% UIs, estimated using Monte Carlo simulations. Abbreviations: DALYs, disability-adjusted life-years; YLDs, years lived with disability; YLLs, years of life lost; ^*^, *p *< 0.05.

### Regional disparities in pancreatic cancer burden, 2021

In 2021, the burden of pancreatic cancer exhibited substantial variation across GBD regions, with clear differences in both magnitude and temporal trends from 1990 to 2021. As shown in S1 Table, high-income areas such as Western Europe, North America, and the High-income Asia Pacific reported the highest age-standardized rates for prevalence, mortality, and DALYs. However, the percentage changes in these regions were relatively modest, suggesting more stabilized control and possibly better access to early diagnosis and treatment. In contrast, rapid increases in disease burden were observed in several low- and middle-income regions. For instance, South Asia and Southeast Asia experienced some of the highest percentage increases in both prevalence and DALY rates, reflecting the growing impact of pancreatic cancer in these transitioning regions. Particularly striking were the sharp rises in Western and Eastern Sub-Saharan Africa, where DALY rates surged by over 80 percent, highlighting the disproportionate burden in resource-limited settings. In East Asia, where China is located, the age-standardized DALY rate reached 137.2 per 100,000 in 2021, accompanied by notable percentage increases in both prevalence and deaths, indicating a significant upward trend over the past three decades. These findings emphasize the urgent need for region-specific strategies in prevention and care.

### Trends of pancreatic cancer burden in China based on Joinpoint regression analysis, 1990–2021

**Fig 3:**
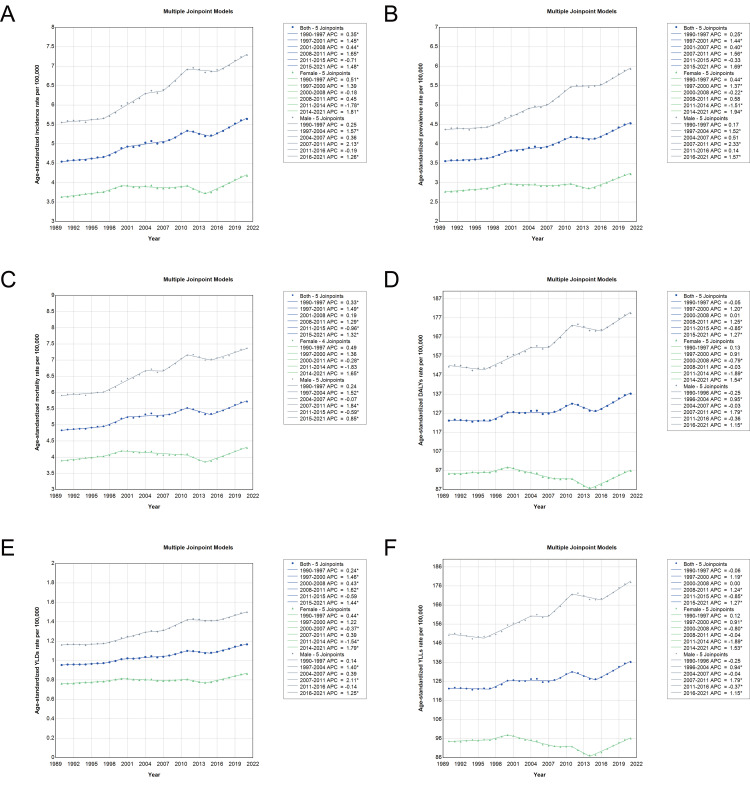
Joinpoint regression analysis of age-standardized rates for pancreatic cancer in China from 1990 to 2021. (A) Age-standardized incidence rates for males (gray line) and females (green line), with the overall population trend represented by the blue line, showing intervals of significant increases. (B) Age-standardized prevalence rates for males, females, and the overall population, illustrating prevalence trends over time. (C) Age-standardized mortality rates for males and females, along with the total population trend, indicating key periods of increase in mortality rates. (D) DALYs for pancreatic cancer, capturing the combined burden across both sexes and the overall population. (E) YLDs for males and females, compared to the overall population, indicating the non-fatal burden over time. (F) YLLs metrics for both sexes, depicting the fatal burden of pancreatic cancer across the population. Abbreviations: DALYs, disability-adjusted life years; YLDs, years lived with disability; YLLs, years of life lost.

Joinpoint regression analysis revealed complex temporal patterns in the burden of pancreatic cancer in China from 1990 to 2021, characterized by distinct phases of trend acceleration and deceleration. As illustrated in Fig 3, the age-standardized incidence, prevalence, and mortality rates generally showed an upward trend, with inflection points reflecting shifts in growth pace. According to S2 Table, incidence rates increased significantly during 1997–2001 (APC 1.45%, 95% CI: 0.69 to 2.21) and again from 2015 to 2021 (APC 1.48%, 95% CI: 1.01 to 1.95), while a temporary decline occurred from 2011 to 2015 (APC −0.71%, 95% CI: −1.62 to 0.20). Prevalence trends followed a similar trajectory, with a sharp increase after 2015 (APC 1.69%, 95% CI: 1.31 to 2.07). Mortality rates mirrored these trends, with a post-2015 rise (APC 1.32%, 95% CI: 0.98 to 1.76). The AAPCs across the full period were 0.72% for incidence, 0.80% for prevalence, and 0.56% for mortality. S3 Table shows that DALY rates also surged from 2015 to 2021 (APC 1.27%, 95% CI: 0.91 to 1.63), following a brief decline from 2011 to 2015 (APC −0.85%, 95% CI: −1.54 to −0.16). YLLs and YLDs displayed similar turning points, with notable increases in the post-2015 period. The AAPC for DALYs was 0.36% for the total population, with higher values observed in men (0.55%) compared to women (0.07%). These findings highlight a period of accelerated burden after 2015, particularly among males, and underscore the importance of interpreting temporal trends through both overall and segmented trend analyses to better understand shifts in disease dynamics and public health priorities.

### Age, period, and cohort effects on pancreatic cancer metrics in China

**Fig 4:**
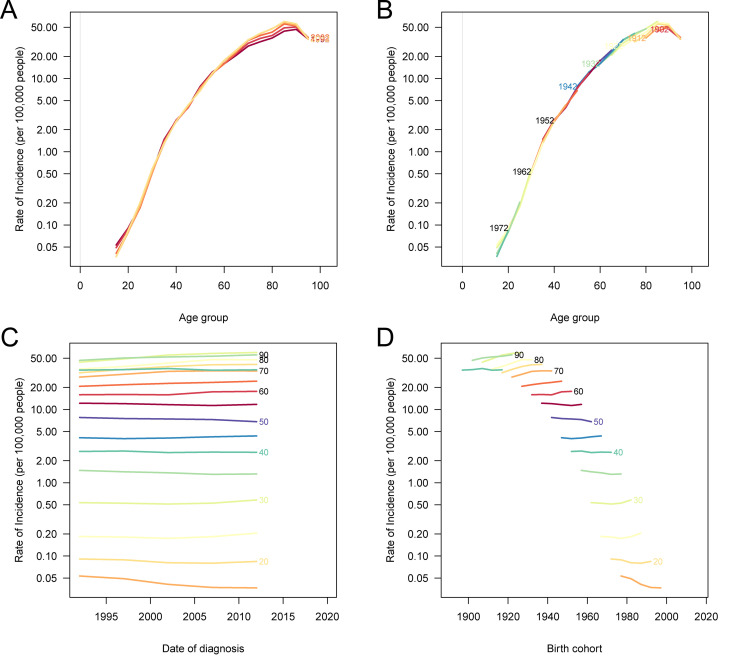
Age, period, and cohort effects on the incidence of pancreatic cancer in China. (A) Age-standardized incidence rates of pancreatic cancer according to time periods; each line connects the age-specific incidence for a 5-year period. (B) Age-standardized incidence rates of pancreatic cancer according to birth cohorts; each line connects the age-specific incidence for a 5-year birth cohort. (C) Period-specific incidence rates of pancreatic cancer according to age groups; each line connects the period-specific incidence for a 5-year age group. (D) Cohort-specific incidence rates of pancreatic cancer according to age groups; each line connects the cohort-specific incidence for a 5-year age group.

The age, period, and cohort analysis of pancreatic cancer metrics in China reveals distinct and escalating patterns over time. Age-specific incidence, prevalence, and DALY rates all increase markedly from the 45–49 age group onward, with this trend persisting across time periods. Analysis by birth cohort shows that successive cohorts, particularly those born after 1950, experience progressively higher age-specific rates, highlighting an increasing burden as these cohorts age. Period-specific rates for incidence, prevalence, and DALYs display an upward trajectory across all age groups, with more recent periods showing the highest rates, especially among older age groups. Cohort-specific analysis also reflects this rising trend, with each successive cohort exhibiting increased rates in later life, underscoring the growing impact of pancreatic cancer in China across generations (Fig 4, S4 and S5 Figs).

### Decomposition analysis of pancreatic cancer incidence, prevalence, and DALYs

**Fig 5:**
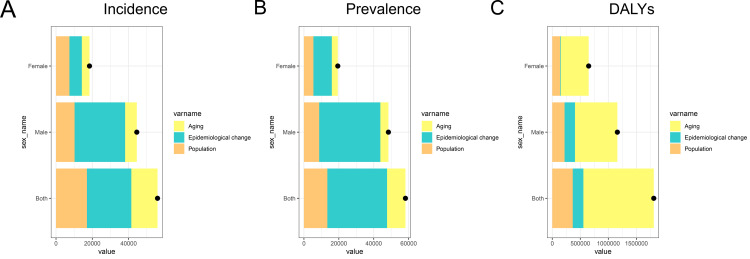
Decomposition analysis of the changes in incidence, prevalence, and DALYs of pancreatic cancer in China, by sex. The contributions of each factor to the total increase are depicted. (A) Decomposition of the increase in pancreatic cancer incidence into aging, epidemiological change, and population growth for both sexes, males, and females. (B) Decomposition of the increase in pancreatic cancer prevalence into aging, epidemiological change, and population growth for both sexes, males, and females. (C) Decomposition of the increase in pancreatic cancer DALYs into aging, epidemiological change, and population growth for both sexes, males, and females.

The decomposition analysis in Fig 5 illustrates the factors contributing to the rise in pancreatic cancer incidence, prevalence, and DALYs in China, segmented by sex. The increase in incidence (Fig 5A) is shown to be driven primarily by epidemiological changes, with additional contributions from population growth and aging, particularly among males. In prevalence (Fig 5B), the aging population emerges as a significant driver, accompanied by epidemiological shifts and population expansion, with males experiencing a more pronounced increase. DALYs (Fig 5C), reflecting the total disease burden, are primarily influenced by aging and epidemiological changes, with males again bearing a greater impact compared to females. This analysis highlights the complex interplay of demographic and epidemiological factors fueling the rising burden of pancreatic cancer in China.

### Projected trends in pancreatic cancer metrics in China using BAPC analysis

**Fig 6:**
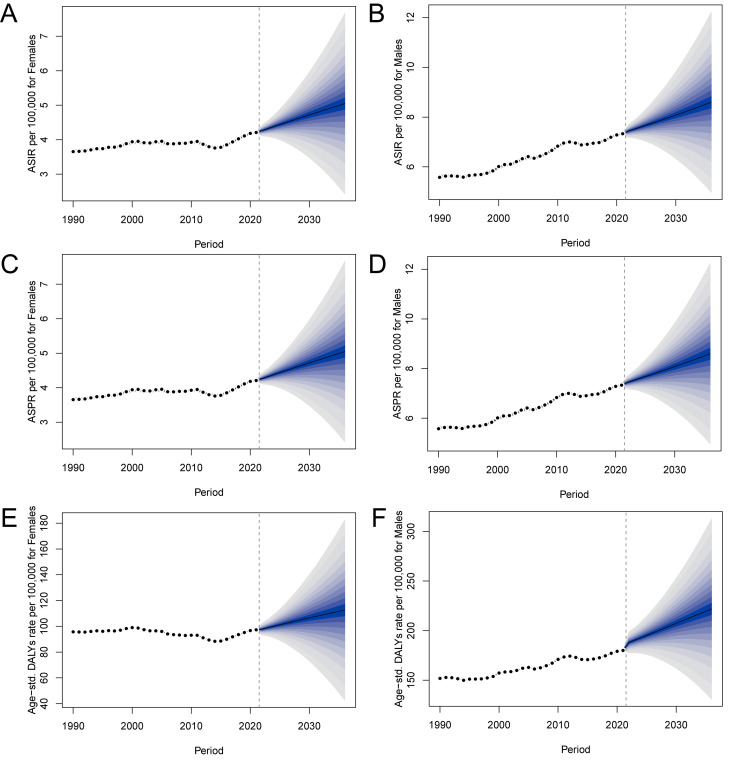
BAPC analysis of pancreatic cancer incidence, prevalence and DALYs in China by sex. (A) BAPC model projections of age-specific pancreatic cancer incidence rates for females. (B) BAPC model projections of age-specific pancreatic cancer incidence rates for males. (C) BAPC model projections of age-specific pancreatic cancer prevalence rates for females. (D) BAPC model projections of age-specific pancreatic cancer prevalence rates for males. (E) BAPC model projections of age-specific pancreatic cancer DALYs rates for females. (F) The projections show the expected trends in DALYs rates across different age groups over time. BAPC, Bayesian age-period-cohort; DALYs, disability-adjusted life years.

BAPC modeling projected a sustained upward trajectory in the age-standardized incidence, prevalence, and DALY rates of pancreatic cancer in China through 2030. As shown in Fig 6, incidence and prevalence rates are expected to rise steadily, particularly among males, suggesting a growing number of new and existing cases due to population aging and persistent exposure to risk factors. Similarly, the DALY rate, which reflects both fatal and non-fatal disease burden, is projected to increase, indicating that the health impact of pancreatic cancer will continue to intensify. These projections reinforce the trends observed in historical data and suggest that without targeted intervention, the societal and clinical burden of pancreatic cancer will worsen over time. The BAPC model highlights the need for proactive public health strategies focused on prevention, early detection, and healthcare resource allocation to counter the projected escalation in disease burden.

## Discussion

In this study, the burden of pancreatic cancer in China was systematically assessed over a three-decade period using data from the GBD Study 2021. The results revealed a continuous and substantial rise in the incidence, prevalence, and DALY rates of pancreatic cancer from 1990 to 2021, with the disease burden in China outpacing global averages. In 2021 alone, over 118,000 new cases and more than 2.9 million DALYs were reported, with age-standardized rates significantly higher among males. Age- and sex-specific analyses indicated that the disease burden was concentrated in older age groups, particularly those over 65 years, and that the majority of health loss was attributable to premature mortality. Temporal trend analyses using joinpoint regression highlighted multiple inflection points, with a notable acceleration in disease burden after 2015. Age-period-cohort modeling further confirmed strong age and cohort effects, with increased risks observed in more recent birth cohorts. Decomposition analysis showed that population aging and epidemiological changes were the main drivers of burden escalation. Projections based on Bayesian APC modeling suggested that incidence, prevalence, and DALY rates will continue to rise through 2030, underscoring the urgency for targeted prevention and control strategies.

Several recent studies have used the GBD framework to examine the global and regional burden of pancreatic cancer, offering essential context for our findings. Amini et al. analyzed GBD 2019 data and identified significant spatial clustering of incidence and mortality, with hotspots in Europe and North America, while highlighting steady global increases in both metrics [24]. Wang et al. and Li et al. expanded this perspective by incorporating GBD 2021 data and forecasting disease burden through 2046–2050, emphasizing the accelerating contribution of modifiable risk factors such as high fasting plasma glucose, high BMI, and smoking, particularly in high-SDI regions and among males [25,26]. Zhao et al. applied age-period-cohort modeling and showed that cohort effects are more pronounced in low- and middle-SDI regions, while period effects are stronger in low-SDI countries, suggesting emerging challenges for health systems in resource-limited settings [27]. Complementing these, Ramai et al. focused on socioeconomic disparities, estimating that the economic impact of pancreatic cancer has risen in tandem with disease burden, further reinforcing the urgency of targeted public health responses [28].

In contrast to these broader analyses, our study provides a focused national perspective based on the most recent GBD 2021 data, revealing that the pancreatic cancer burden in China is increasing at a pace that outstrips global averages. Compared to our previous work on stomach cancer using the same methodology [9], which showed a declining trend driven by effective prevention, pancreatic cancer demonstrates a markedly different epidemiologic trajectory, underlining the unique challenges posed by this malignancy. Our findings emphasize the value of localized, site-specific analyses to inform disease control strategies, complement global overviews, and facilitate tailored public health interventions. Huang and his colleagues highlighted that diabetes and obesity, two conditions that are more commonly found in males, serve as substantial risk factors for pancreatic cancer [29]. The differences in risk factor profiles, healthcare systems, and demographic trends across regions further contextualize these findings. In high-income regions, such as Western Europe and North America, advanced healthcare systems facilitate early detection and management, but lifestyle factors like obesity and alcohol consumption remain key drivers of incidence. In contrast, in low-income regions, limited access to healthcare infrastructure exacerbates the disease burden due to delayed diagnosis and higher mortality rates.

In China, periods of rapid urbanization and socioeconomic development, as identified through Joinpoint regression analysis (specifically from 1997 to 2001 and from 2008 to 2011), coincided with substantial lifestyle changes, including increased tobacco consumption and greater intake of high-fat, energy-dense diets. McGuigan et al. highlighted the association between such transitions and heightened cancer risk, which aligns with our observations [30]. Additionally, demographic transitions, particularly population aging, have played a pivotal role in the increasing burden of pancreatic cancer. Older individuals in both developed and emerging economies are more likely to develop the disease due to biological aging processes and cumulative exposure to carcinogenic risk factors. The sharp rise in incidence, prevalence, and mortality among men aged 80 to 85 years may be explained by immune system decline, higher rates of comorbidities such as diabetes and chronic pancreatitis, and long-term exposure to tobacco and alcohol, which are more prevalent among older men in China. Delayed diagnosis and limited access to curative treatment options in this age group may also contribute to elevated mortality. According to our decomposition analysis, aging contributed more substantially to the rise in pancreatic cancer incidence among women, whereas epidemiological changes, particularly those related to behavioral and environmental exposures, had a stronger influence among men. This sex-specific pattern is consistent with findings by Ilic and Ilic [8], who reported that men are more likely to engage in high-risk behaviors such as smoking and excessive alcohol consumption, both of which are well-established contributors to pancreatic carcinogenesis.

The projections from our BAPC model indicate a continuous rise in pancreatic cancer incidence through 2030, underscoring the urgency for targeted public health interventions. These projections align with previous studies [31,32], which similarly forecasted increasing pancreatic cancer rates in Europe due to comparable demographic and lifestyle factors. The growing incidence is linked to several modifiable risk factors, including smoking, which doubles the risk due to tobacco carcinogens causing DNA damage [33], and heavy alcohol consumption, particularly in men, which increases risk through chronic pancreatitis [34]. Obesity, especially abdominal obesity, contributes through inflammation and insulin resistance [35,36], while long-standing diabetes promotes tumor growth through hyperglycemia and elevated insulin levels [37]. Dietary habits, such as high intake of red and processed meats and low intake of fruits and vegetables, also play a critical role [10,38]. Conversely, diets rich in fruits, vegetables, and whole grains reduce risk through antioxidants and fiber, which mitigate inflammation and improve insulin sensitivity [39].

Genetic predispositions, including BRCA1, BRCA2, and PALB2 mutations, further elevate risk, particularly when combined with environmental and lifestyle factors [40]. In China, studies like Chen et al.‘s multicenter case-control analysis have highlighted smoking, heavy alcohol use, and chronic pancreatitis as significant risk factors [41]. The increasing prevalence of diabetes in China has also been closely linked to the rising pancreatic cancer rates [42,43]. Wang et al. emphasized that lifestyle changes, including high-fat diets and physical inactivity driven by urbanization, have exacerbated the cancer burden [44]. The rapid urbanization and associated lifestyle changes in China have led to increased exposure to risk factors such as high-fat diets and sedentary behavior, exacerbating the risk of developing pancreatic cancer [45–47]. Cohort effects observed in our study indicate that more recent birth cohorts have higher pancreatic cancer incidence rates, suggesting early exposure to risk factors. Yang et al. similarly noted that shifts in reproductive patterns, diet, and physical activity among younger generations may lead to increased cancer rates as they age [48–50].

The differences in pancreatic cancer diagnosis, treatment, and risk factors between China and Western countries highlight the need for region-specific prevention strategies. In Western countries, advanced healthcare systems support earlier detection, but rising obesity, diabetes, and alcohol consumption remain significant challenges. In China, rapid urbanization has driven lifestyle changes such as increased tobacco use, high-fat diets, and sedentary behavior, contributing to the rising pancreatic cancer burden. Effective prevention in China should focus on addressing modifiable risk factors through public health campaigns promoting healthy lifestyles, tobacco cessation, and diabetes management, alongside strengthening primary healthcare and early screening programs for high-risk populations. Bridging healthcare access disparities in rural areas and adopting systematic cancer registries and multidisciplinary treatment approaches seen in Western countries could further improve outcomes. Collaborative research into genetic and environmental risk factors would refine prevention strategies, emphasizing the importance of tailored, multifaceted approaches to reduce the global burden of pancreatic cancer.

Despite the strengths of our study, several limitations should be acknowledged. The GBD data used in our analysis are derived from multiple sources, including cancer registries, vital registration systems, and verbal autopsy reports, which may vary in quality and completeness across regions and time periods. As a result, potential biases such as underreporting, misclassification, and inconsistencies in diagnostic or death coding may have affected the accuracy of the estimates, particularly in earlier years or rural areas. Additionally, while the GBD modeling framework (e.g., CODEm and DisMod-MR 2.1) is designed to address missing data and ensure internal consistency, the reliance on statistical estimation introduces uncertainty that may not fully capture local epidemiological dynamics. Moreover, the national-level data may obscure substantial regional heterogeneity within China, potentially limiting the generalizability of our findings across provinces and population subgroups. The dataset also lacks detailed clinical information, including the proportion of cases at different disease stages, rates of surgical intervention, and types of systemic treatments. This absence restricts our ability to evaluate how disease progression and treatment access influence observed burden trends. Furthermore, our BAPC model projections are based on historical patterns and assume that past trends will continue, without incorporating potential shifts in risk factor prevalence, health policy, diagnostic technology, or treatment efficacy. Lastly, the study was limited by the lack of individual-level data on key determinants such as smoking history, body mass index, diabetes status, early-life exposures, and socioeconomic conditions. This limitation hinders a more precise evaluation of how lifestyle and environmental factors have contributed to changes in pancreatic cancer burden. Future research should focus on collecting high-resolution data, including clinical staging and treatment pathways, to better understand the roles of disease biology, healthcare access, and gender-specific risk profiles in shaping long-term trends and guiding targeted interventions.

## Conclusions

In conclusion, the burden of pancreatic cancer in China has risen significantly over the past three decades, demanding urgent public health measures. Addressing modifiable risk factors such as smoking, obesity, and diabetes through targeted prevention strategies is essential. Improving early detection and diagnostic capabilities can contribute to better survival outcomes. Future research should focus on region-specific data to identify the underlying causes of temporal trends and gender disparities. Investigating biological mechanisms and tracking lifestyle and environmental exposures through longitudinal cohort studies can help guide tailored interventions. Importantly, future studies should also adopt a bottom-up approach, beginning with local research teams working within communities to explore the social and cultural determinants of pancreatic cancer. These determinants are often obscured in national-level data and are essential for designing context-specific public health policies and ensuring equitable access to prevention, early diagnosis, and high-quality care. Strengthened collaboration among public health authorities, researchers, and healthcare providers will be crucial to mitigate the growing impact of pancreatic cancer in China.

## Supporting information

S1 Fig Age and sex distribution of DALYs, YLDs, and YLLs rates of pancreatic cancer in China in 2021. (A) Age-specific and gender-specific number of DALYs due to pancreatic cancer. (B) Age-specific and gender-specific rate of DALYs due to pancreatic cancer per 100,000 people. (C) Age-specific and gender-specific number of YLDs due to pancreatic cancer. (D) Age-specific and gender-specific rate of YLDs due to pancreatic cancer per 100,000 people. (E) Age-specific and gender-specific number of YLLs due to pancreatic cancer. (F) Age-specific and gender-specific rate of YLLs due to pancreatic cancer per 100,000 people. Abbreviations: DALYs, disability-adjusted life years; YLDs, years lived with disability; YLLs, years of life lost.
(PDF)

S2 Fig Age-specific numbers and crude rates of pancreatic cancer burden in China in 1990 and 2021. (A) Number and crude incidence rate per 100,000 population, (B) number and crude prevalence rate per 100,000 population, (C) number and crude mortality rate per 100,000 population, (D) number and crude DALY rate per 100,000 population, (E) number and crude YLD rate per 100,000 population, and (F) number and crude YLL rate per 100,000 population, presented by 5-year age groups. Abbreviations: DALYs, disability-adjusted life years; YLDs, years lived with disability; YLLs, years of life lost.
(PDF)

S3 Fig Trends in age-standardized pancreatic cancer burden rates globally and in China, 1990–2021. (A) Global trends in age-standardized incidence rate, mortality rate, prevalence rate, DALYs rate, YLDs rate, and YLLs rate for pancreatic cancer from 1990 to 2021. (B) Trends in age-standardized pancreatic cancer burden rates in China during the same period. Abbreviations: DALYs, disability-adjusted life years; YLDs, years lived with disability; YLLs, years of life lost.
(PDF)

S4 Fig Age, period, and cohort effects on the prevalence of pancreatic cancer in China. (A) Age-standardized prevalence rates of pancreatic cancer according to time periods; each line connects the age-specific mortality for a 5-year period. (B) Age-standardized prevalence rates of pancreatic cancer according to birth cohorts; each line connects the age-specific mortality for a 5-year birth cohort. (C) Period-specific prevalence rates of pancreatic cancer according to age groups; each line connects the period-specific mortality for a 5-year age group. (D) Cohort-specific prevalence rates of pancreatic cancer according to age groups; each line connects the cohort-specific mortality for a 5-year age group.
(PDF)

S5 Fig Age, period, and cohort effects on the DALYs of pancreatic cancer in China. (A) Age-standardized DALY rates of pancreatic cancer according to time periods; each line connects the age-specific mortality for a 5-year period. (B) Age-standardized DALY rates of pancreatic cancer according to birth cohorts; each line connects the age-specific mortality for a 5-year birth cohort. (C) Period-specific DALY rates of pancreatic cancer according to age groups; each line connects the period-specific mortality for a 5-year age group. (D) Cohort-specific DALY rates of pancreatic cancer according to age groups; each line connects the cohort-specific mortality for a 5-year age group. DALY, disability-adjusted life year.
(PDF)

S1 Table Prevalent cases, deaths and DALYs for pancreatic cancer in 2021, and percentage change in ASRs per 100000, by GBD region, from 1990 to 2021. (DOCX)

S2 Table Joinpoint regression analysis of trends in age-standardized incidence, prevalence, mortality rates (per 100,000) by sex for pancreatic cancer in China, 1990–2021. (DOCX)

S3 Table Joinpoint regression analysis of trends in age-standardized DALYs, YLDs, and YLLs rates (per 100,000) by sex for pancreatic cancer in China, 1990–2021. (DOCX)
