# Supplementary figures and images for: Rising burden of pancreatic cancer in China: Trends, drivers, and future projections
Source: PLoS One. 2025 Jul 1;20(7):e0327009. doi: 10.1371/journal.pone.0327009 (PMC12212494; doi:10.1371/journal.pone.0327009)

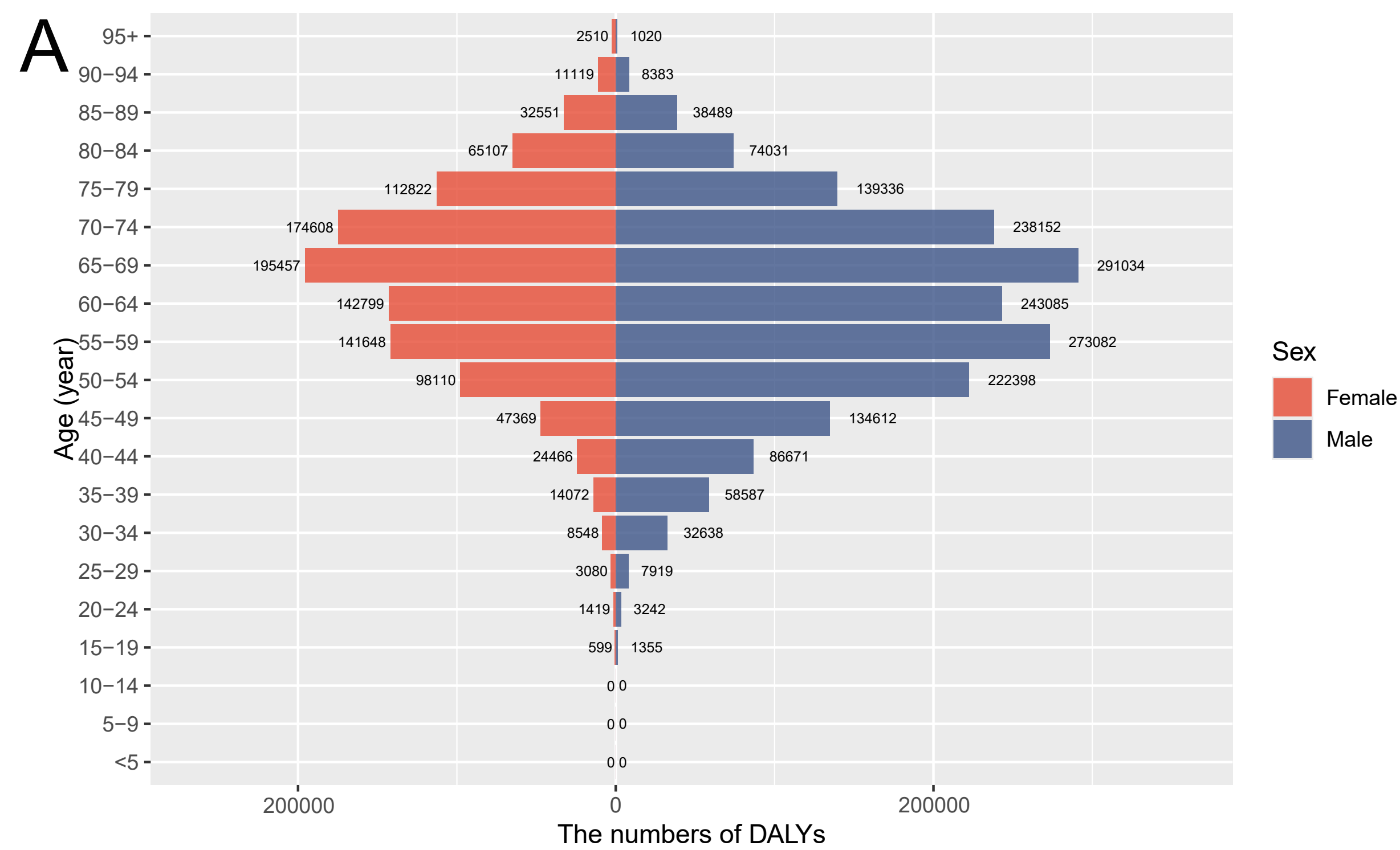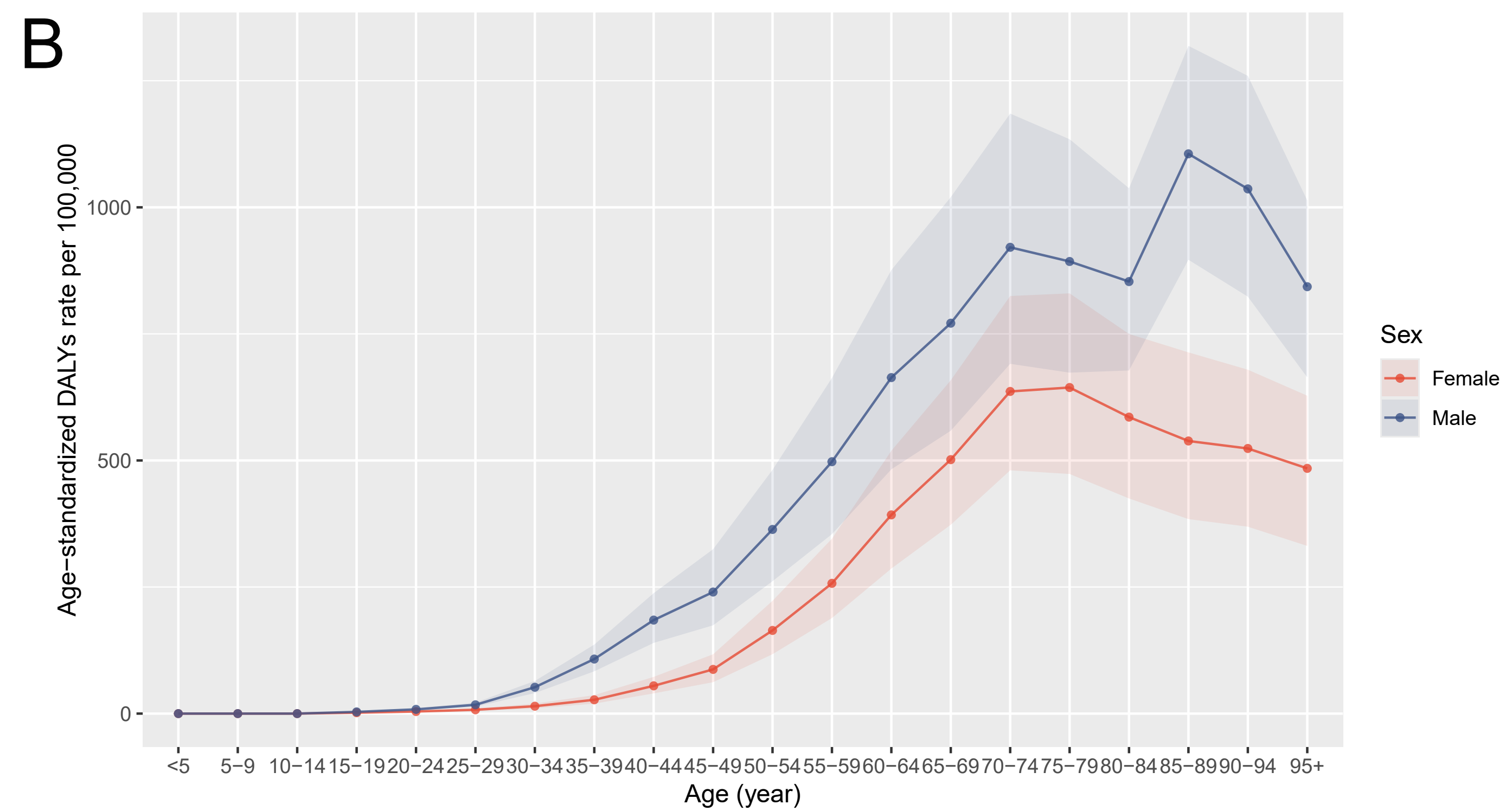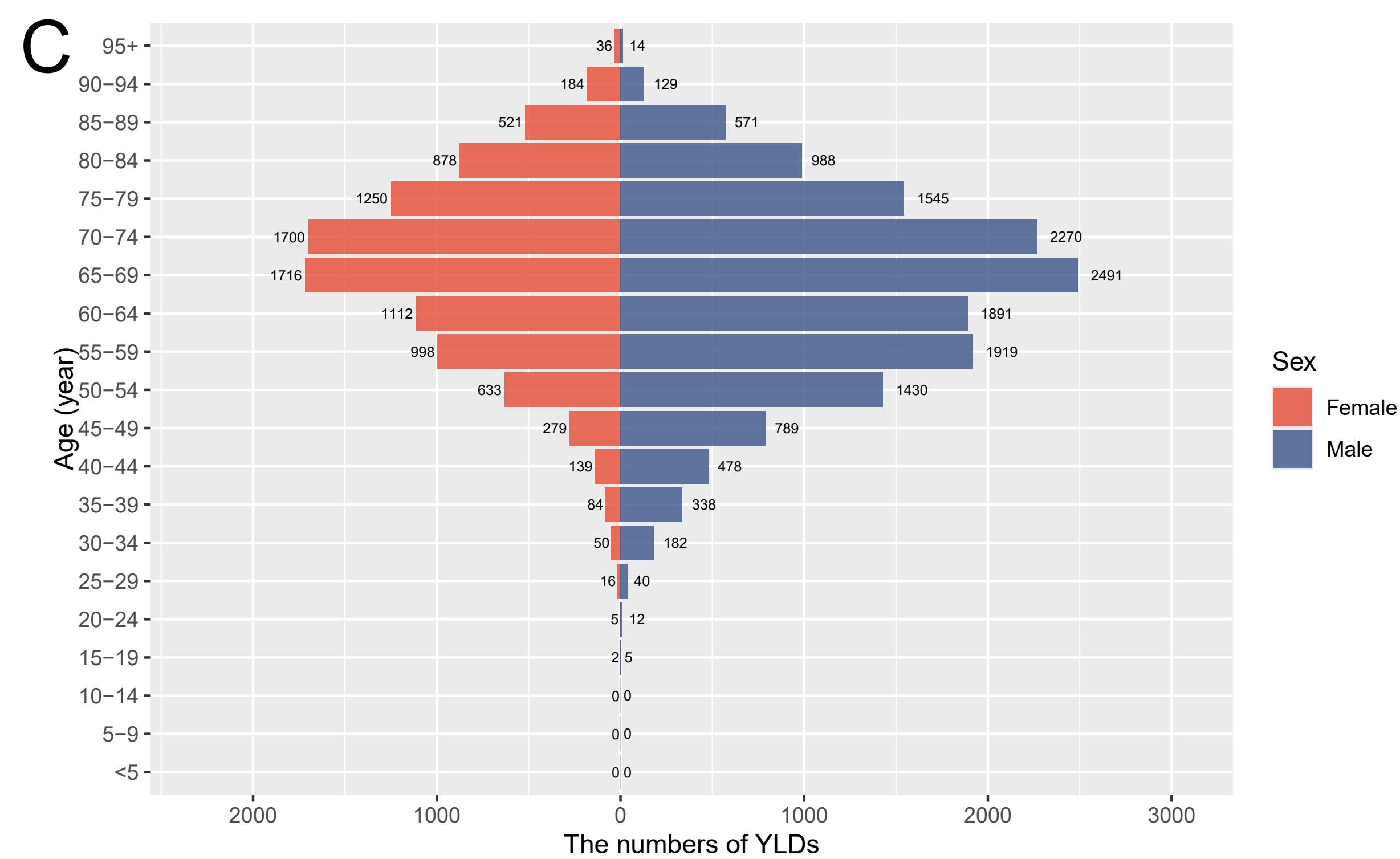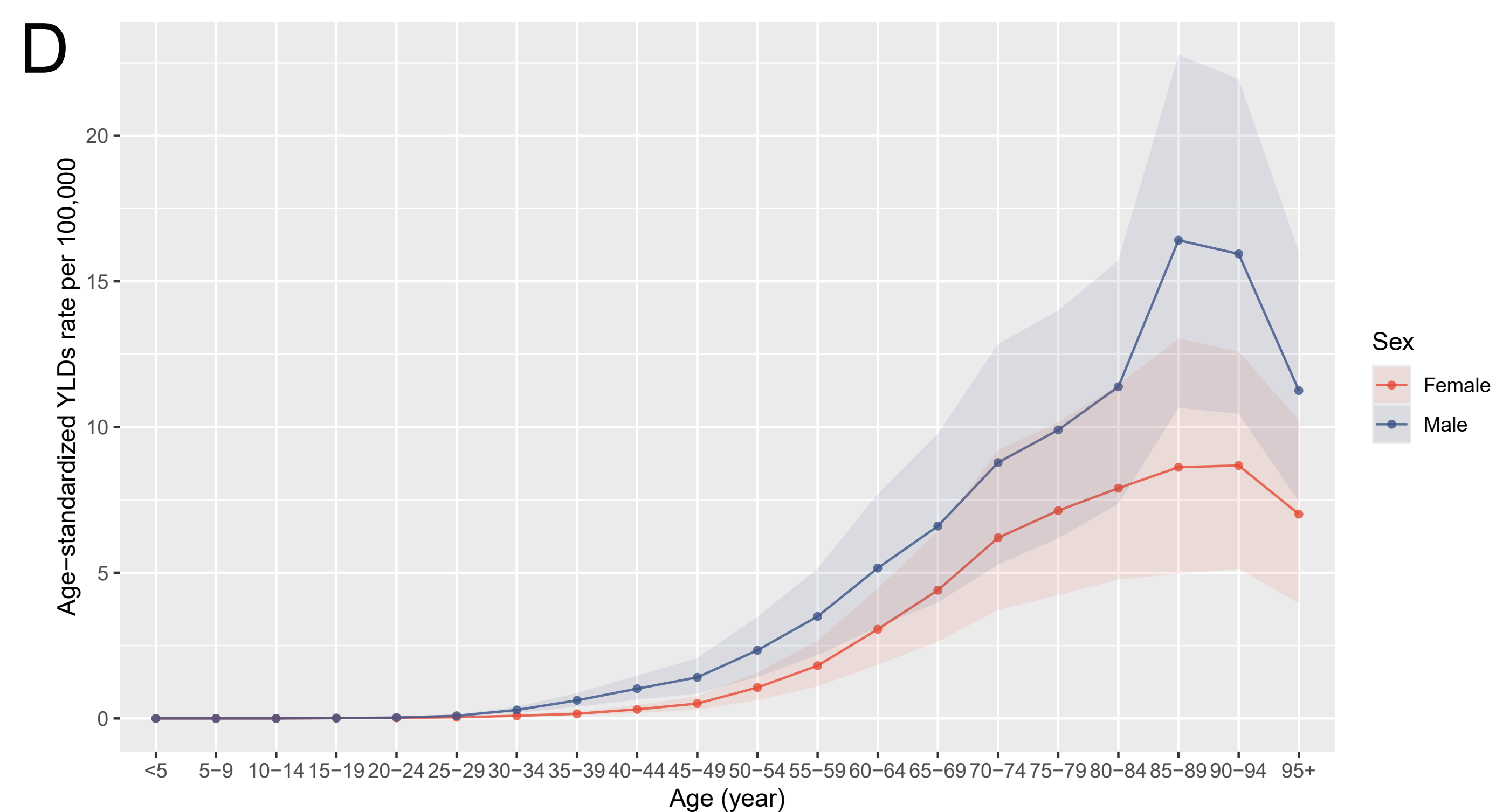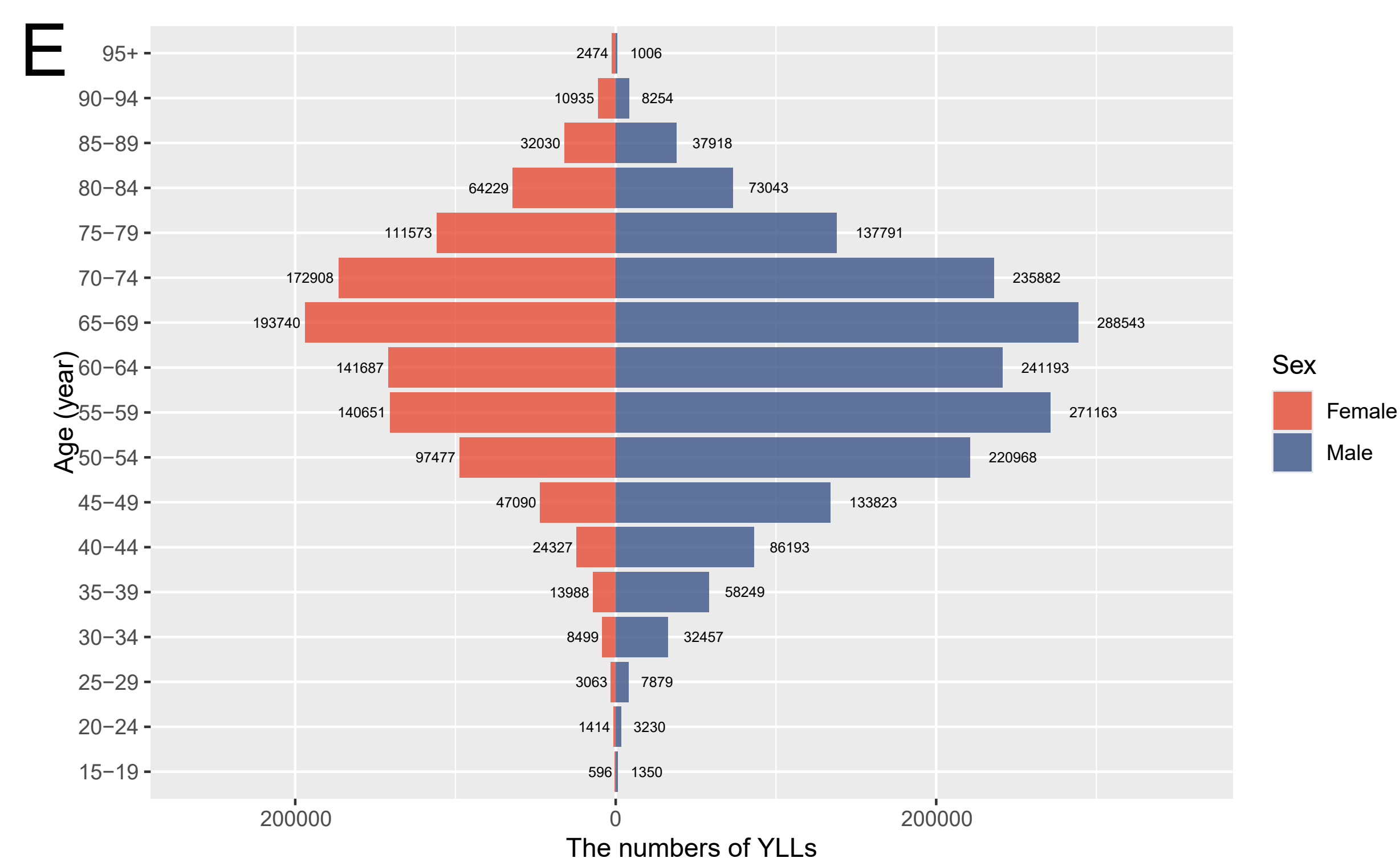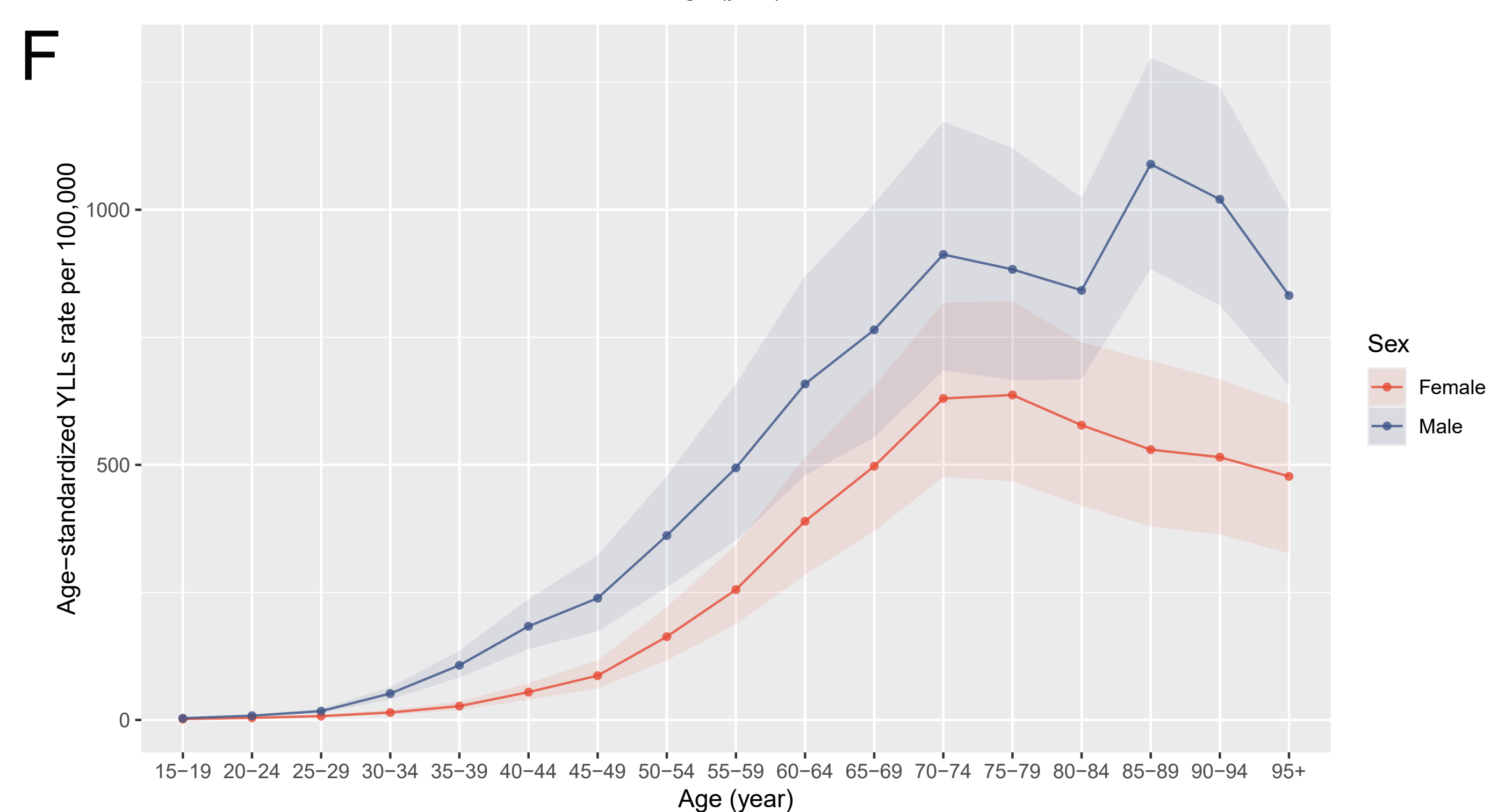

Supplement: S1 Fig — (A) Age-specific and gender-specific number of DALYs due to pancreatic cancer. (B) Age-specific and gender-specific rate of DALYs due to pancreatic cancer per 100,000 people. (C) Age-specific and gender-specific number of YLDs due to pancreatic cancer. (D) Age-specific and gender-specific rate of YLDs due to pancreatic cancer per 100,000 people. (E) Age-specific and gender-specific number of YLLs due to pancreatic cancer. (F) Age-specific and gender-specific rate of YLLs due to pancreatic cancer per 100,000 people. Abbreviations: DALYs, disability-adjusted life years; YLDs, years lived with disability; YLLs, years of life lost. (PDF) [file pone.0327009.s001.pdf]

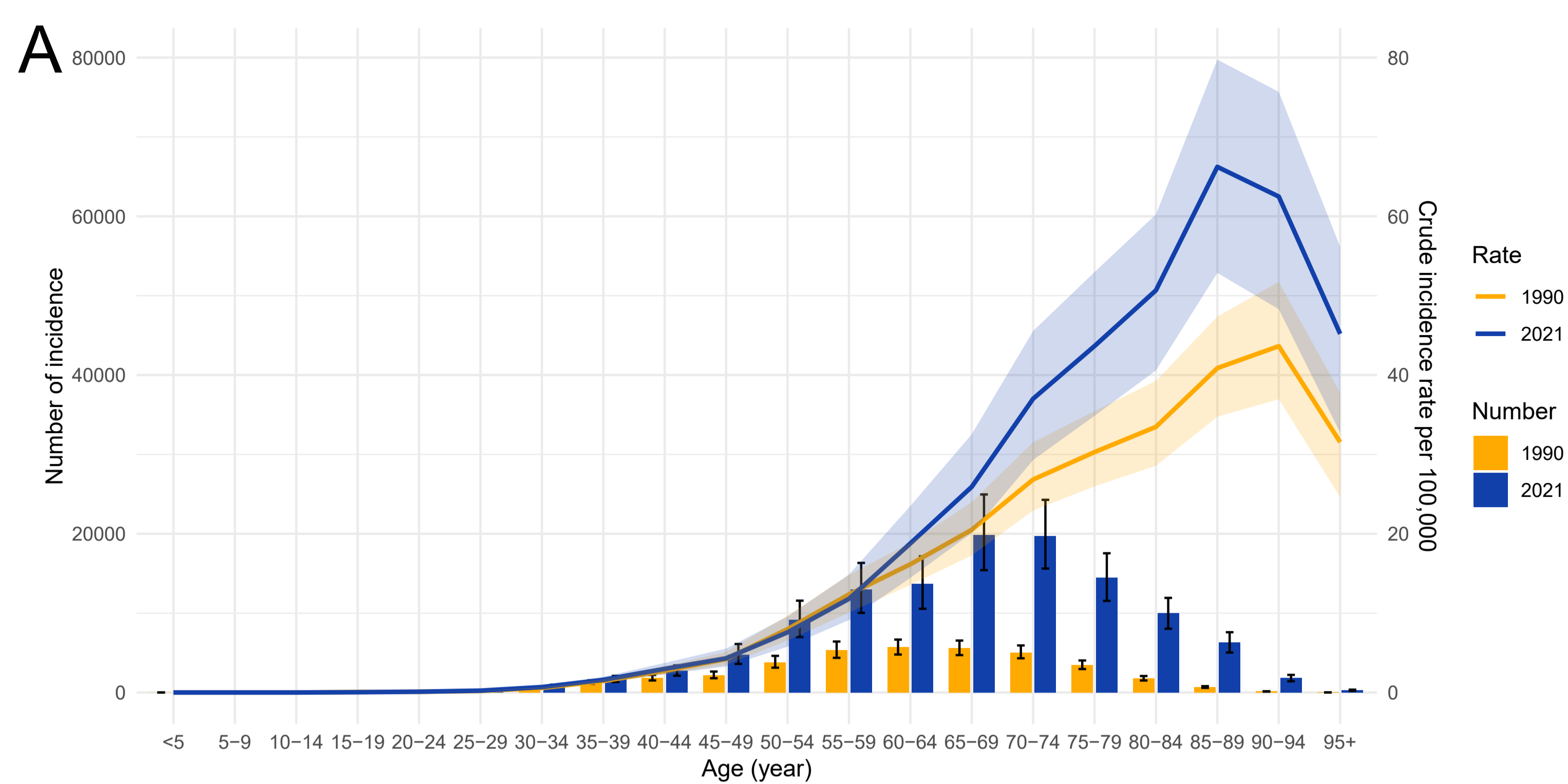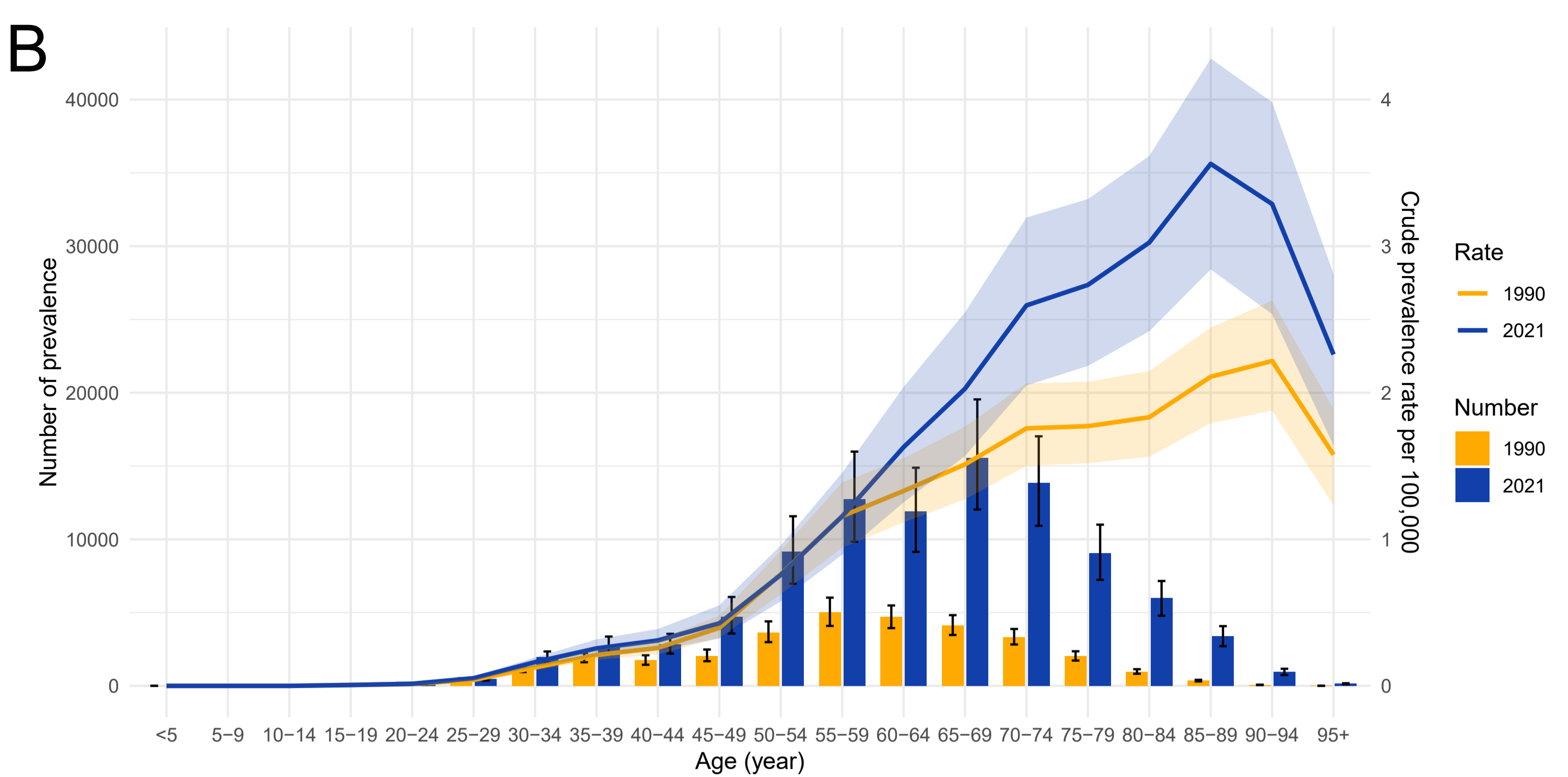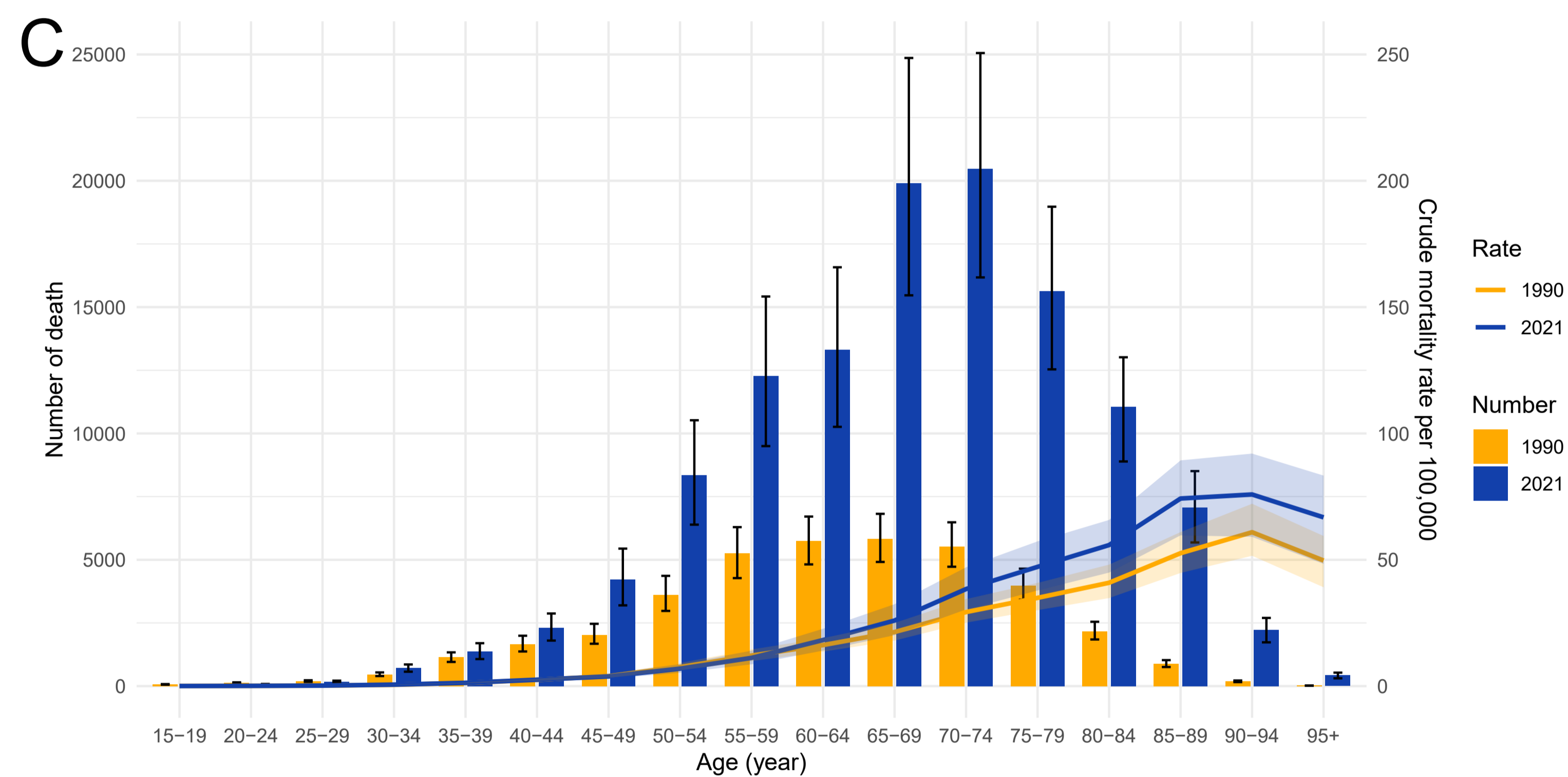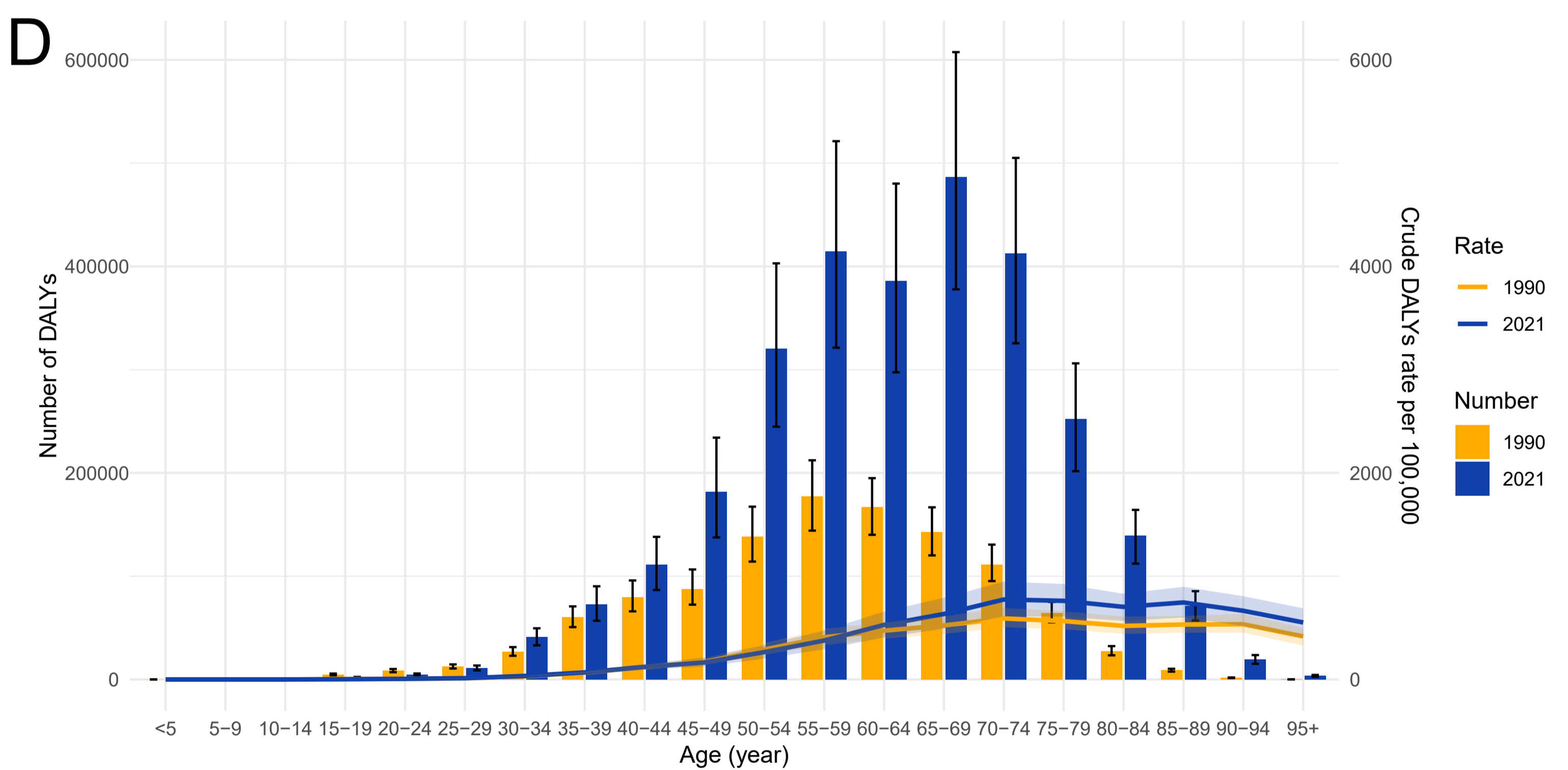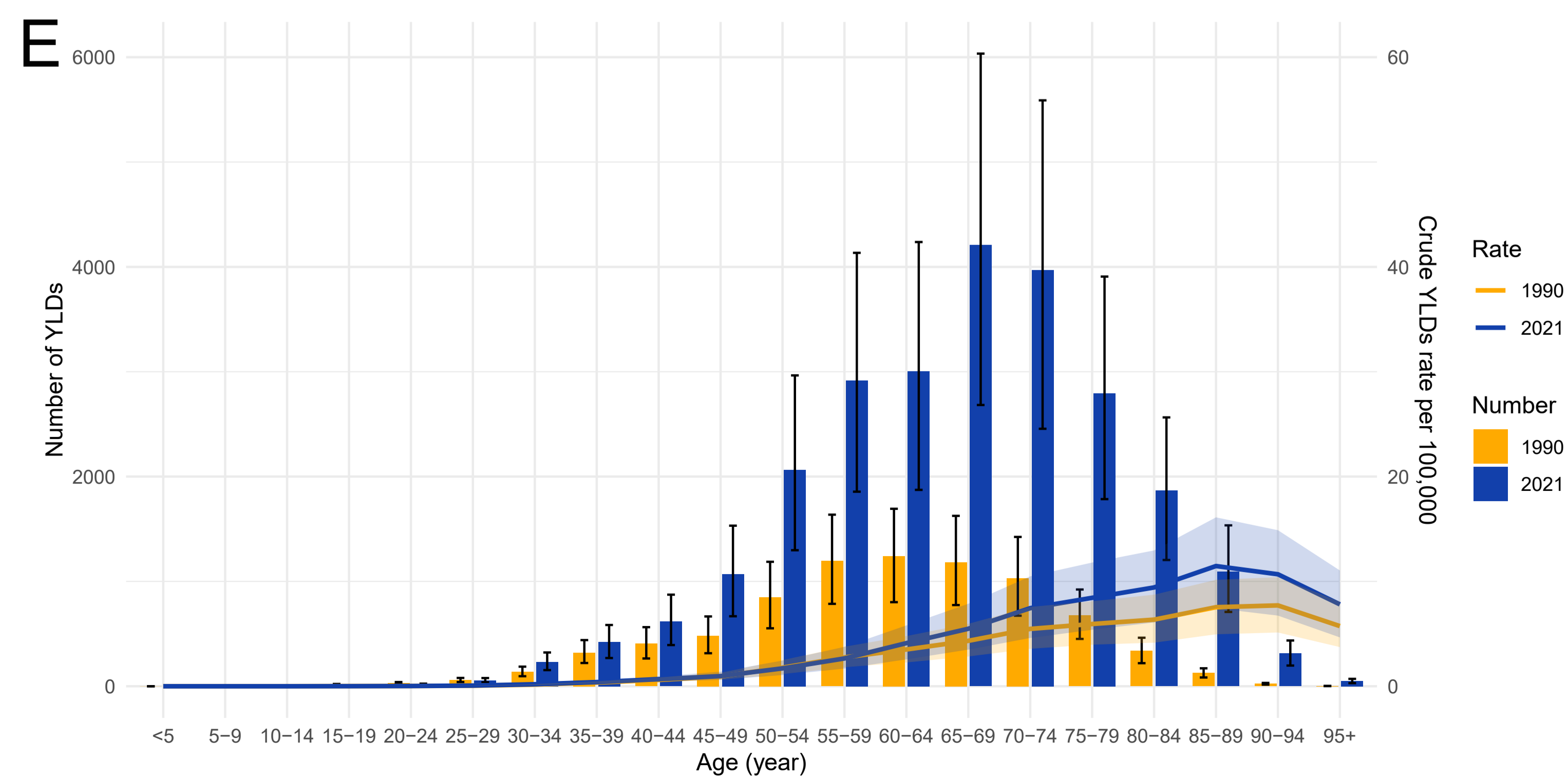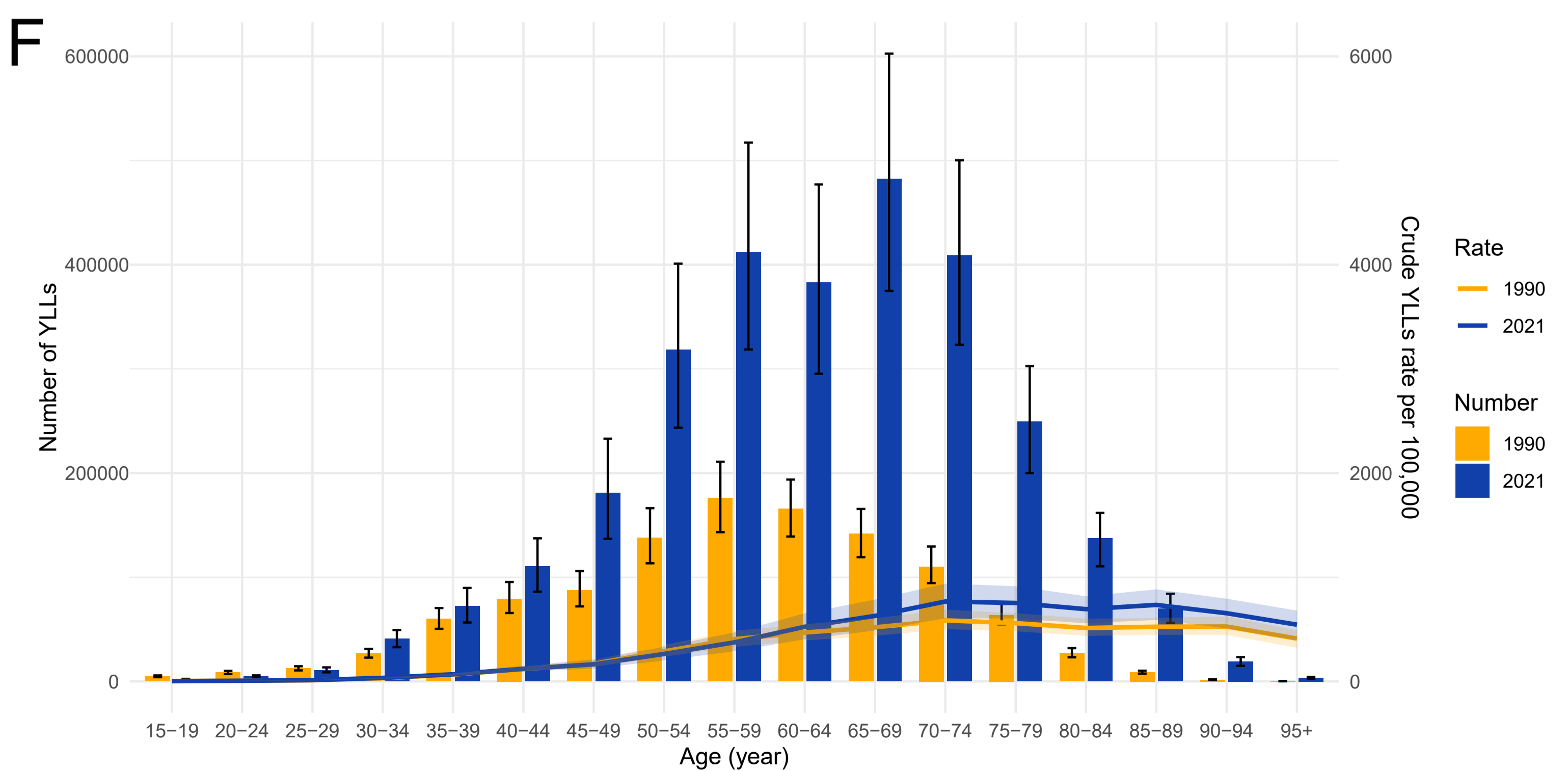

Supplement: S2 Fig — (A) Number and crude incidence rate per 100,000 population, (B) number and crude prevalence rate per 100,000 population, (C) number and crude mortality rate per 100,000 population, (D) number and crude DALY rate per 100,000 population, (E) number and crude YLD rate per 100,000 population, and (F) number and crude YLL rate per 100,000 population, presented by 5-year age groups. Abbreviations: DALYs, disability-adjusted life years; YLDs, years lived with disability; YLLs, years of life lost. (PDF) [file pone.0327009.s002.pdf]

A

1990–2021 Global Age-standardized Rate

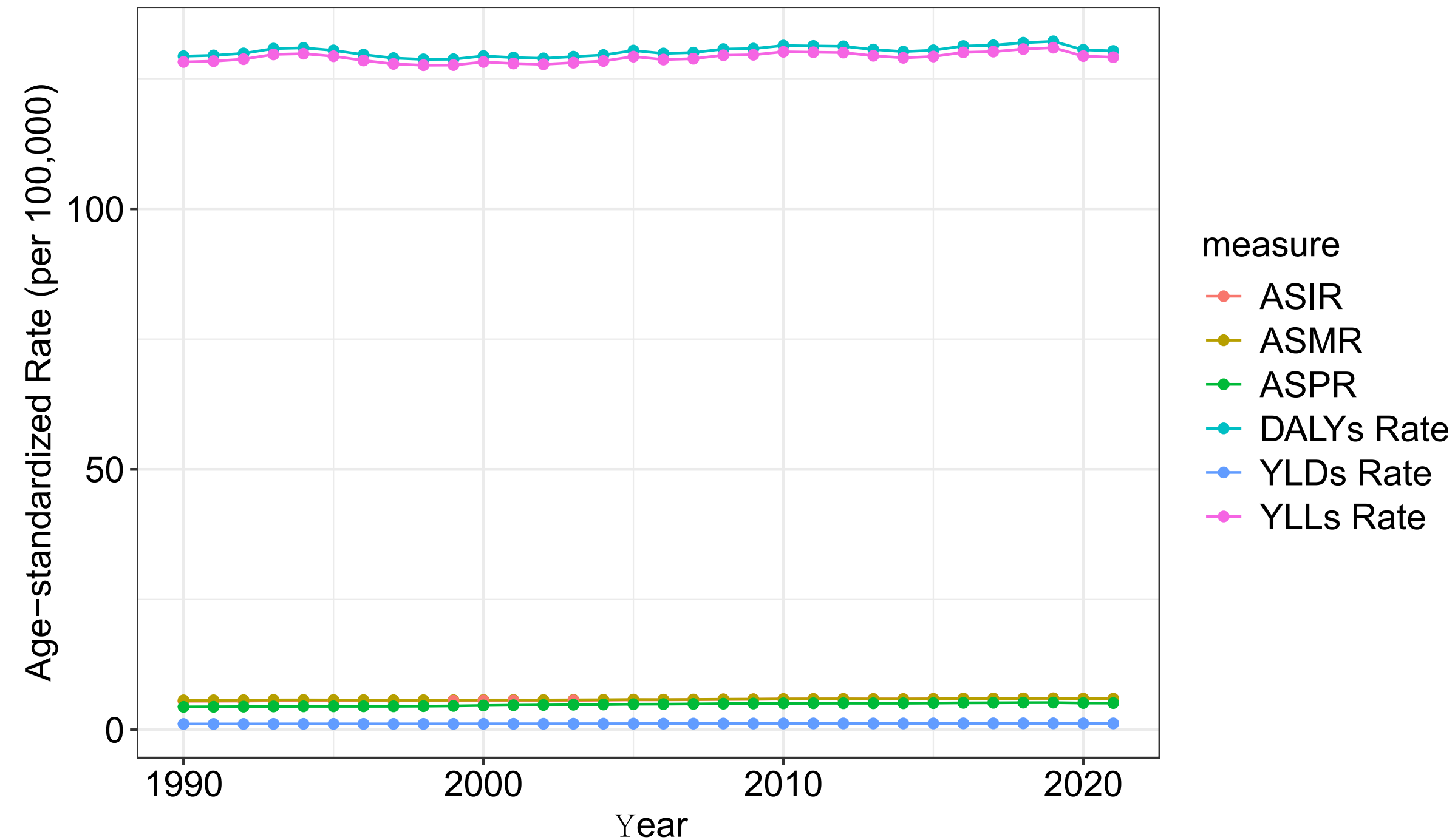

B

1990–2021 China Age-standardized Rate

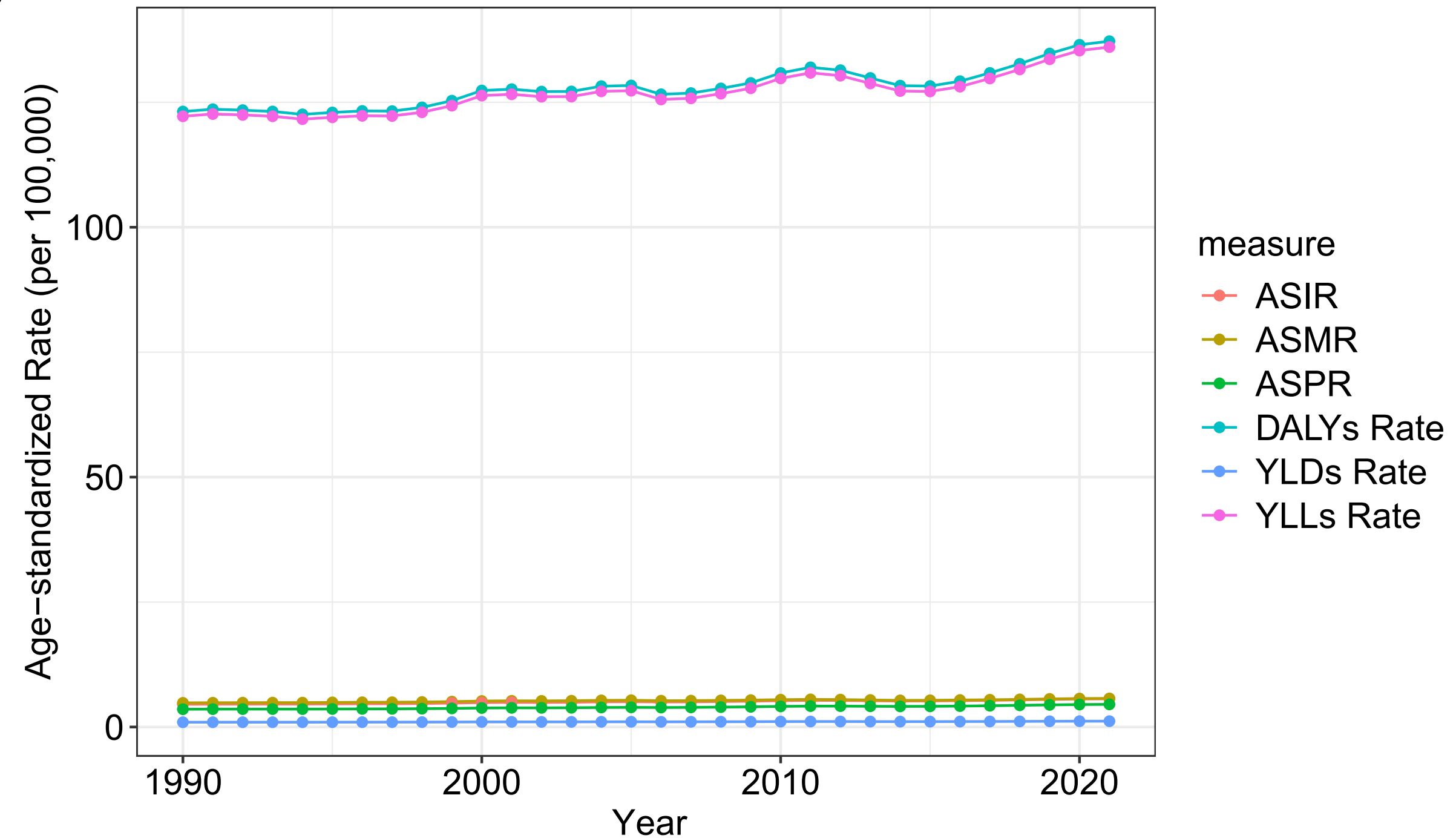

Supplement: S3 Fig — (A) Global trends in age-standardized incidence rate, mortality rate, prevalence rate, DALYs rate, YLDs rate, and YLLs rate for pancreatic cancer from 1990 to 2021. (B) Trends in age-standardized pancreatic cancer burden rates in China during the same period. Abbreviations: DALYs, disability-adjusted life years; YLDs, years lived with disability; YLLs, years of life lost. (PDF) [file pone.0327009.s003.pdf]

**A**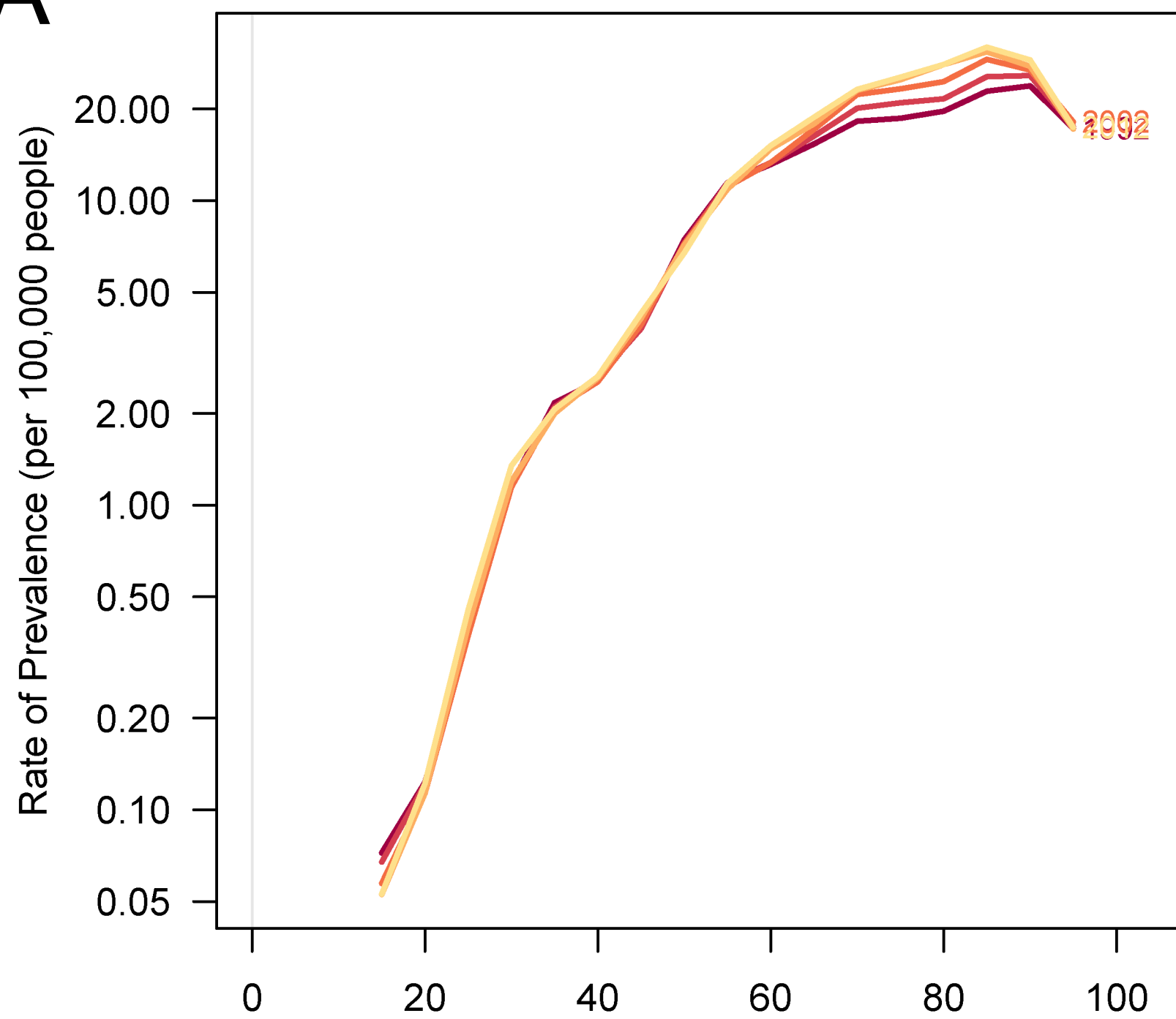**B**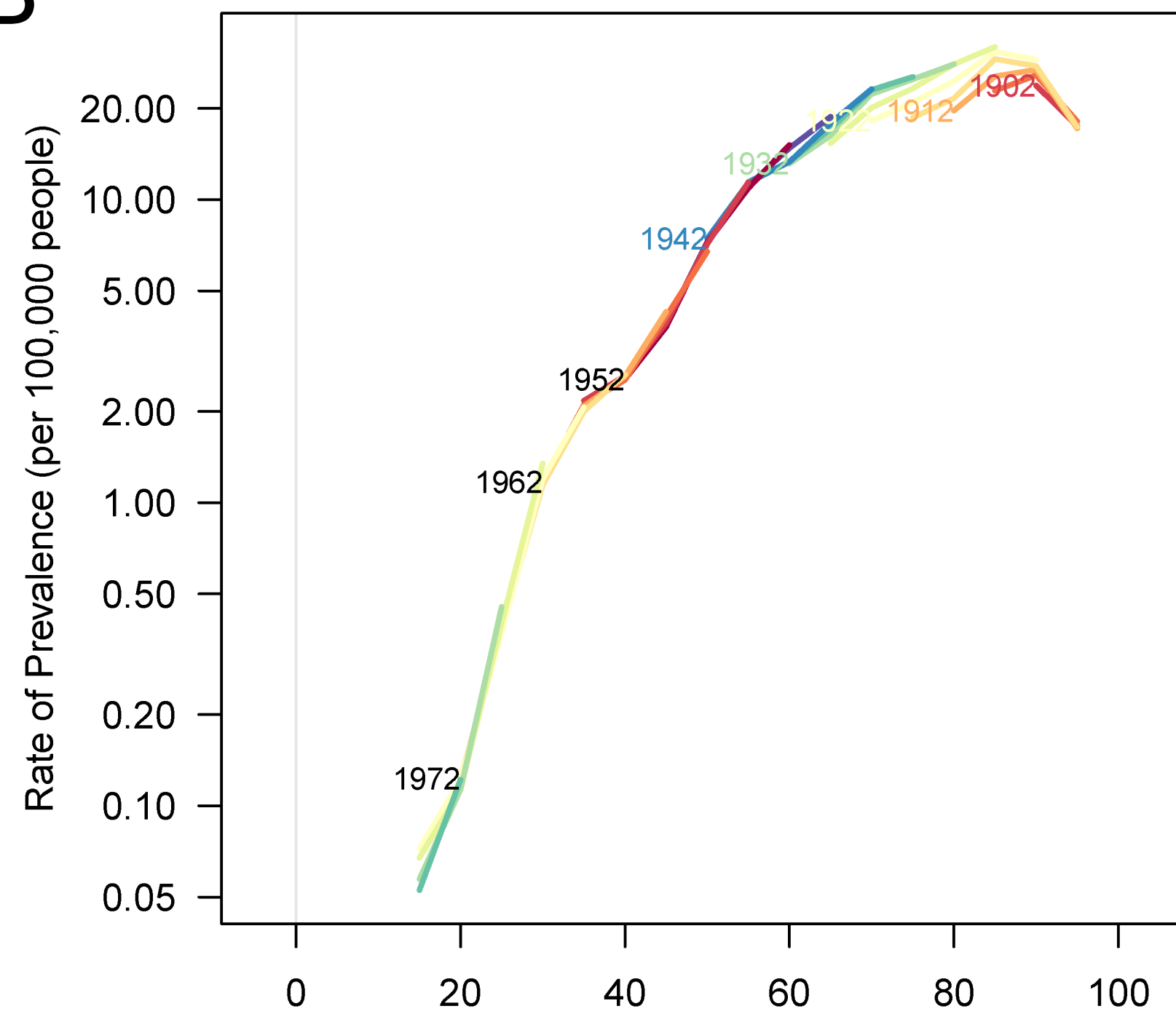**C**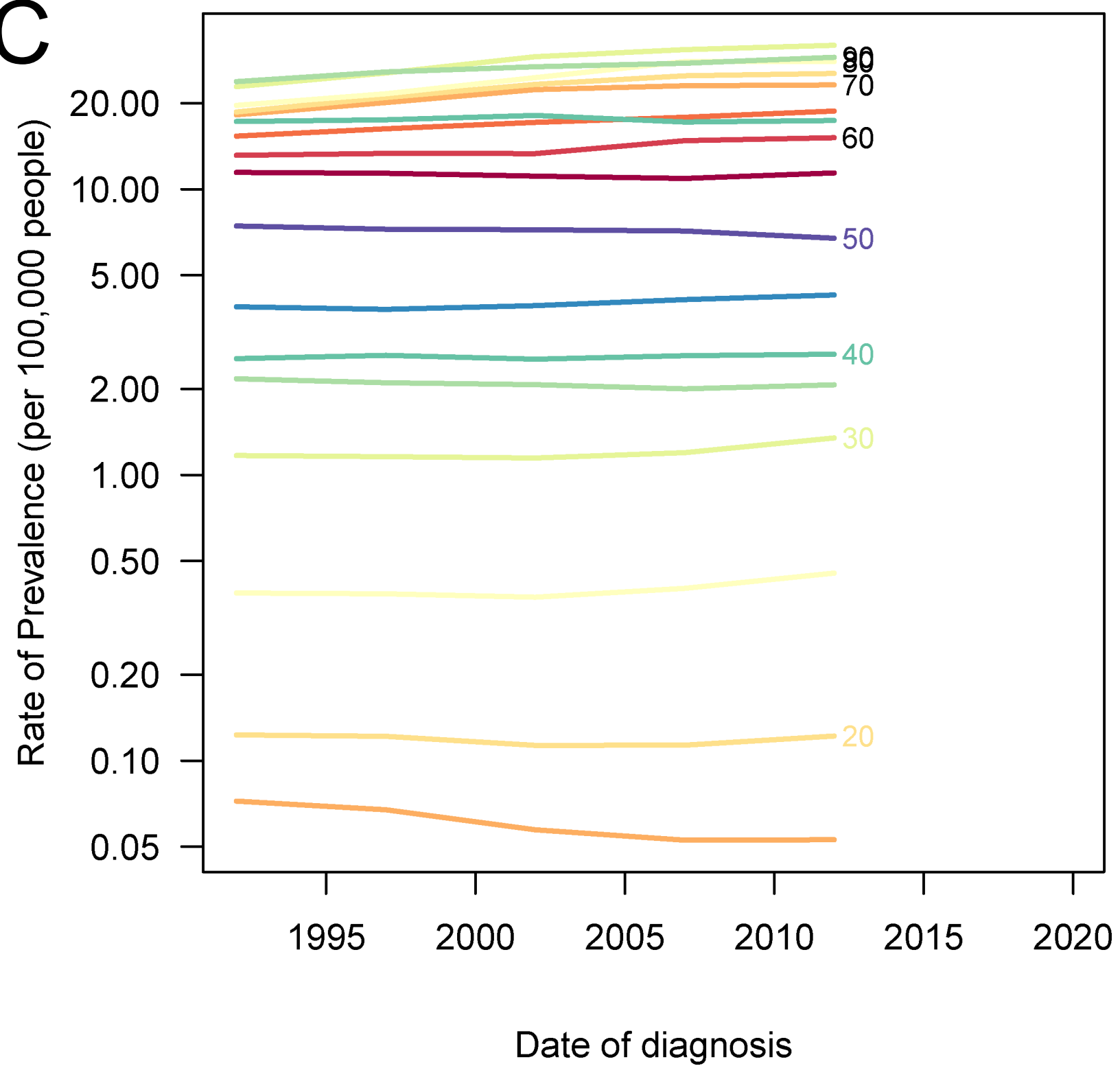**D**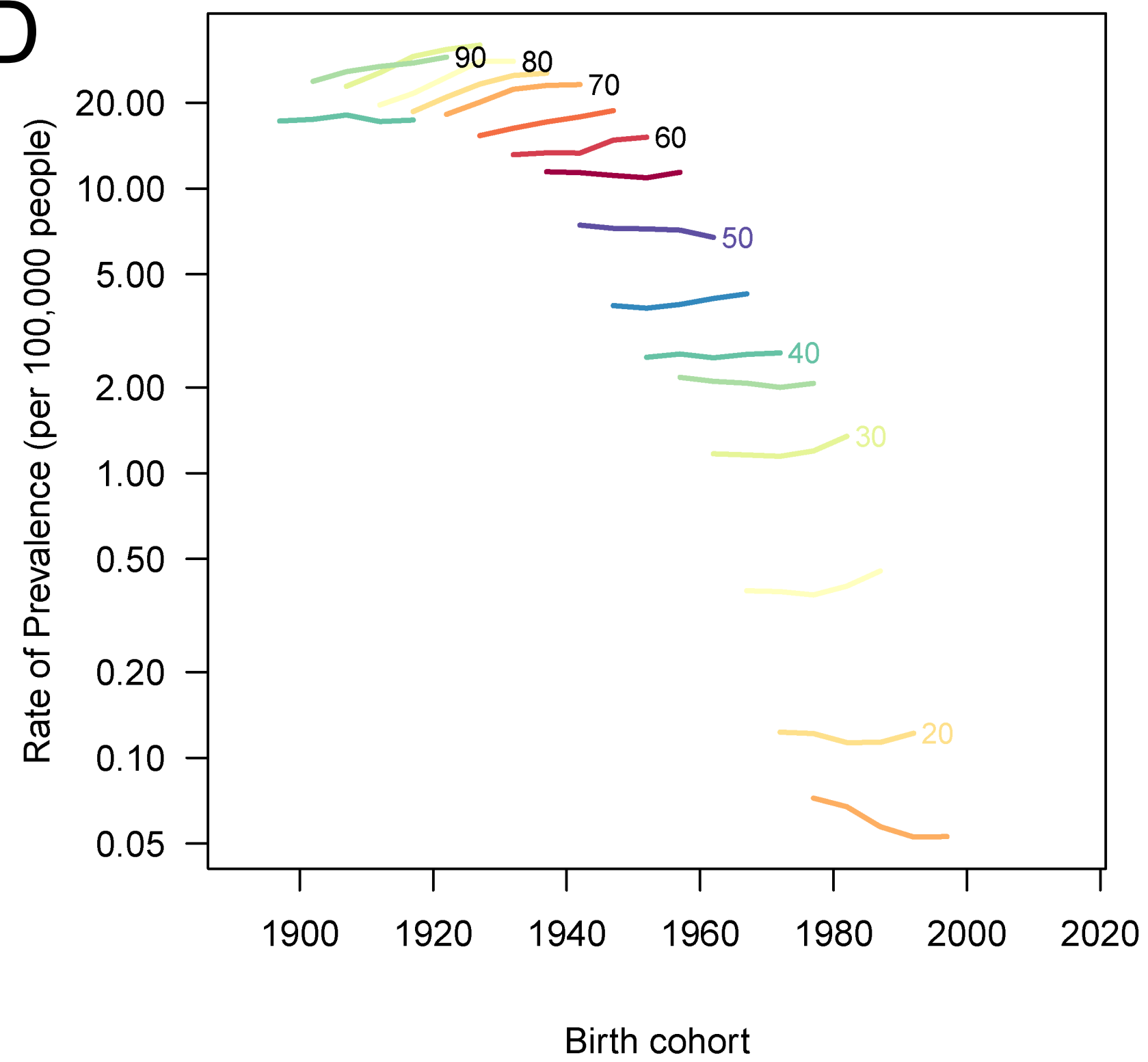

Supplement: S4 Fig — (A) Age-standardized prevalence rates of pancreatic cancer according to time periods; each line connects the age-specific mortality for a 5-year period. (B) Age-standardized prevalence rates of pancreatic cancer according to birth cohorts; each line connects the age-specific mortality for a 5-year birth cohort. (C) Period-specific prevalence rates of pancreatic cancer according to age groups; each line connects the period-specific mortality for a 5-year age group. (D) Cohort-specific prevalence rates of pancreatic cancer according to age groups; each line connects the cohort-specific mortality for a 5-year age group. (PDF) [file pone.0327009.s004.pdf]

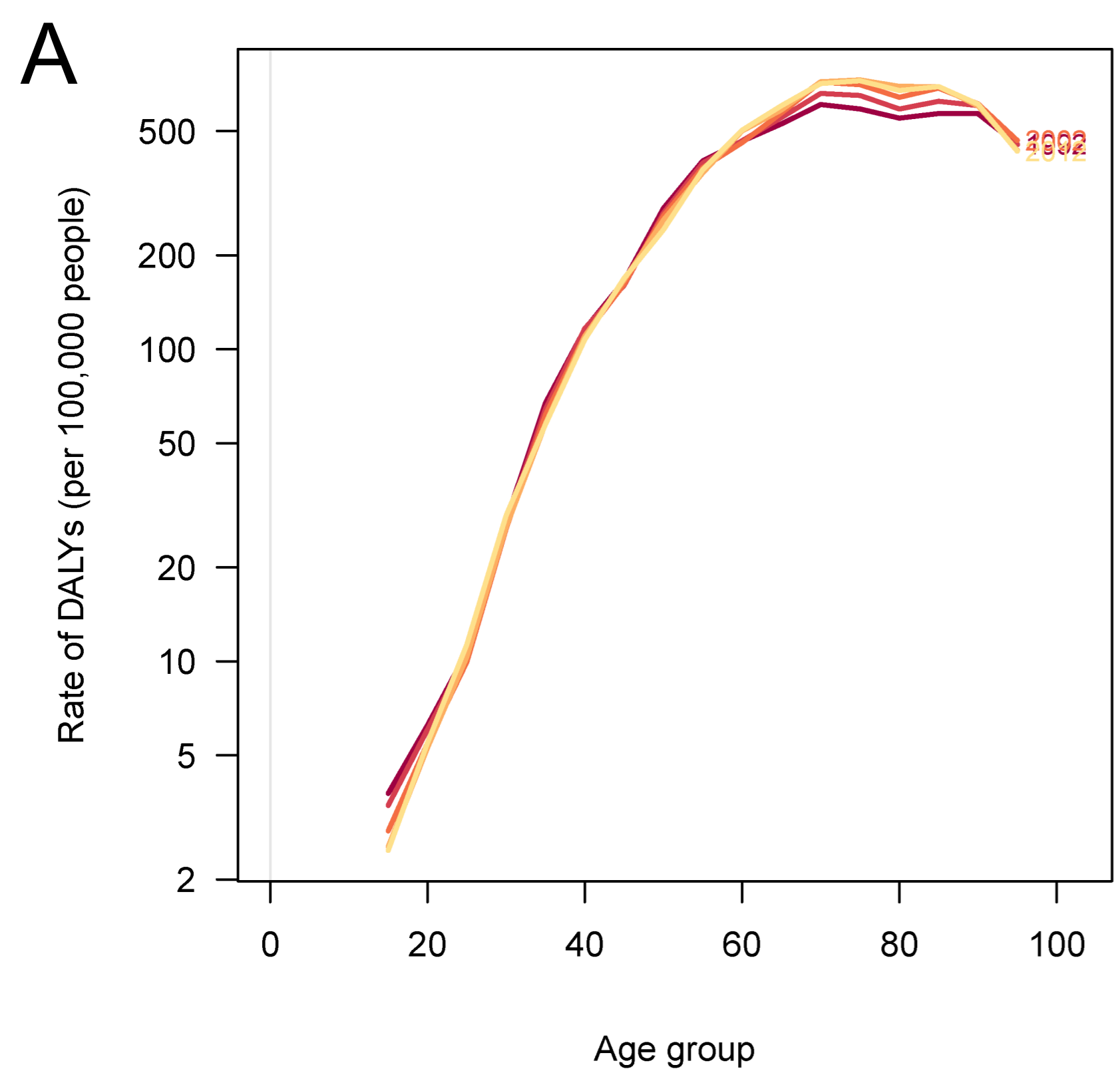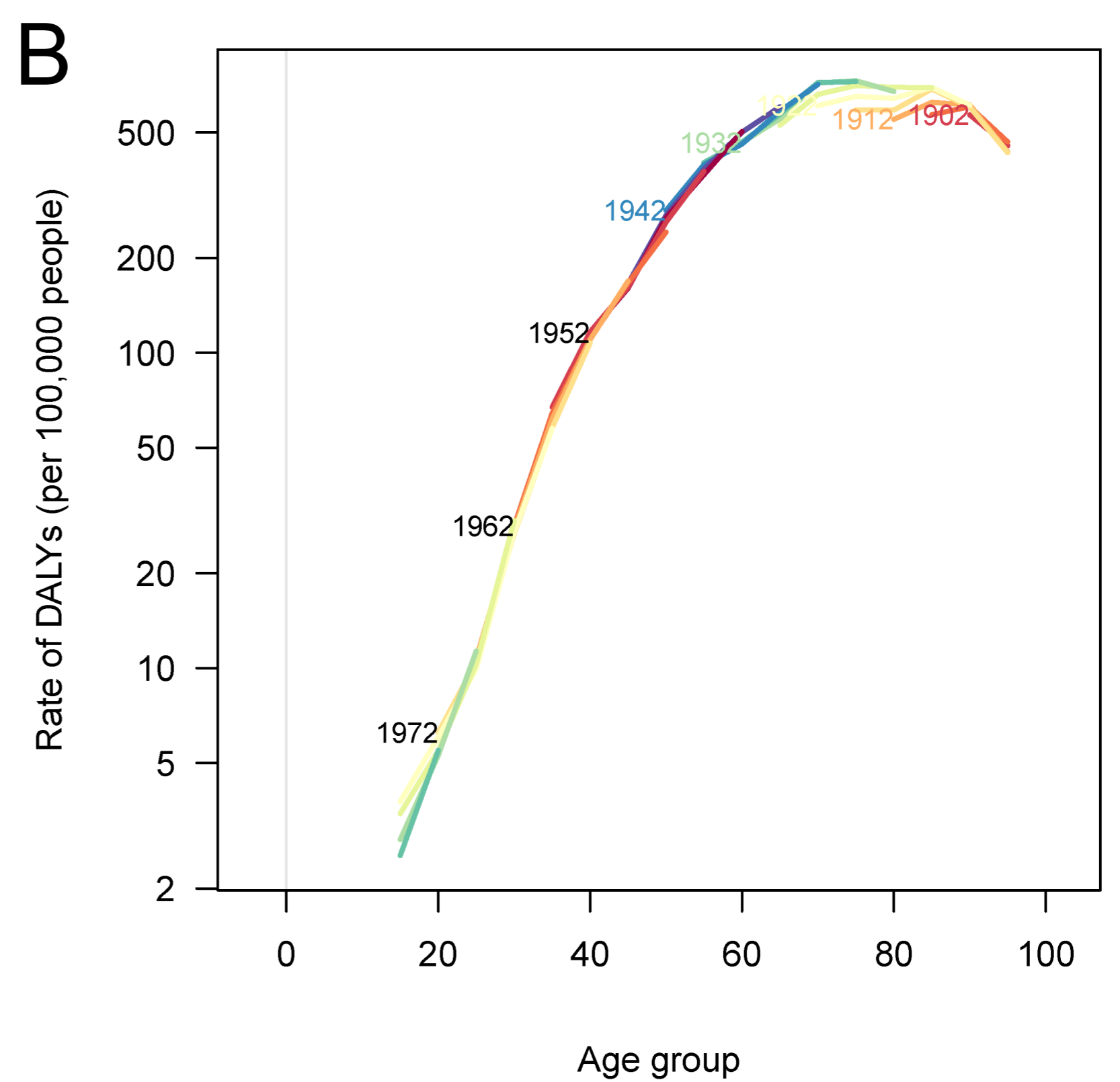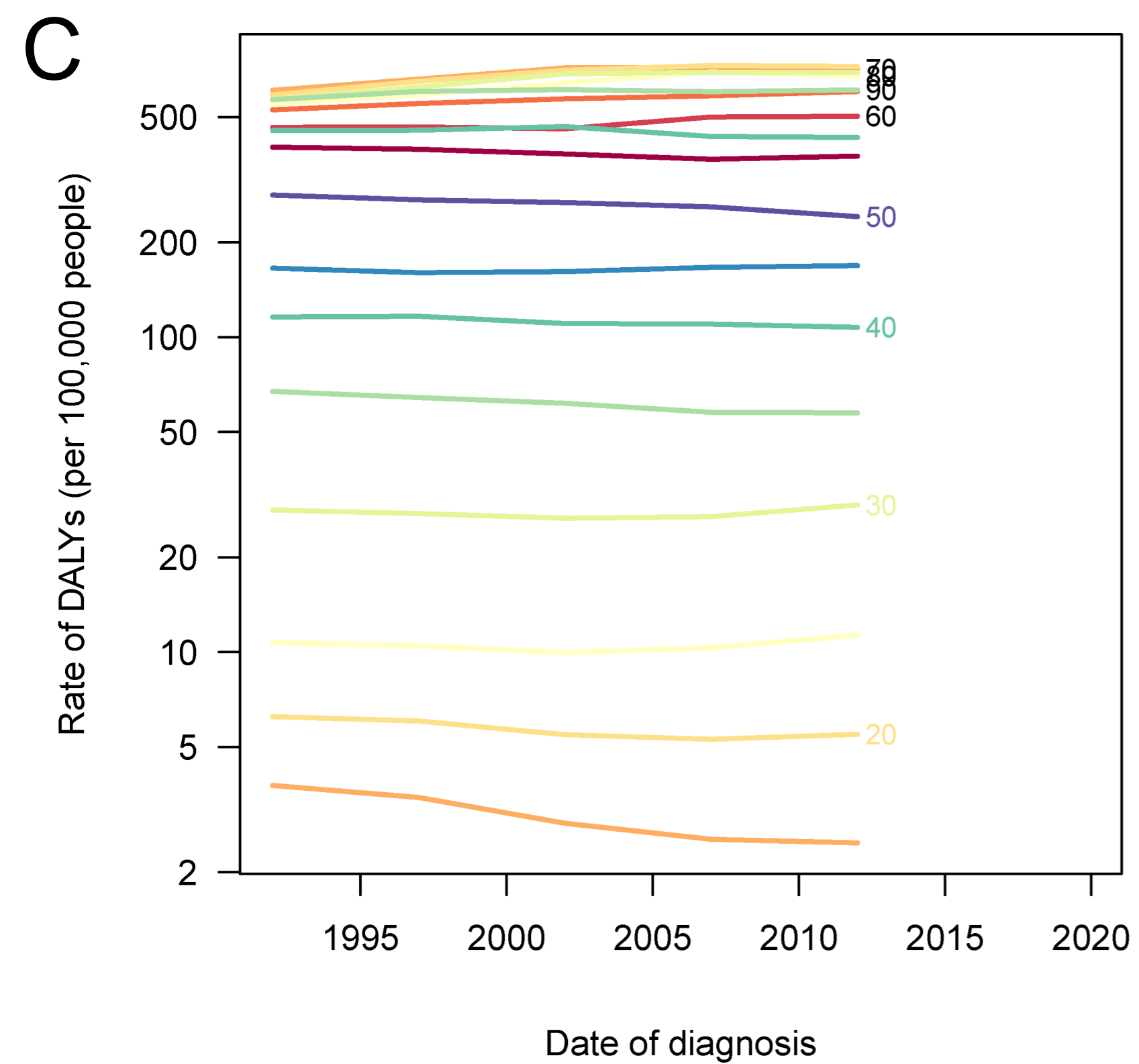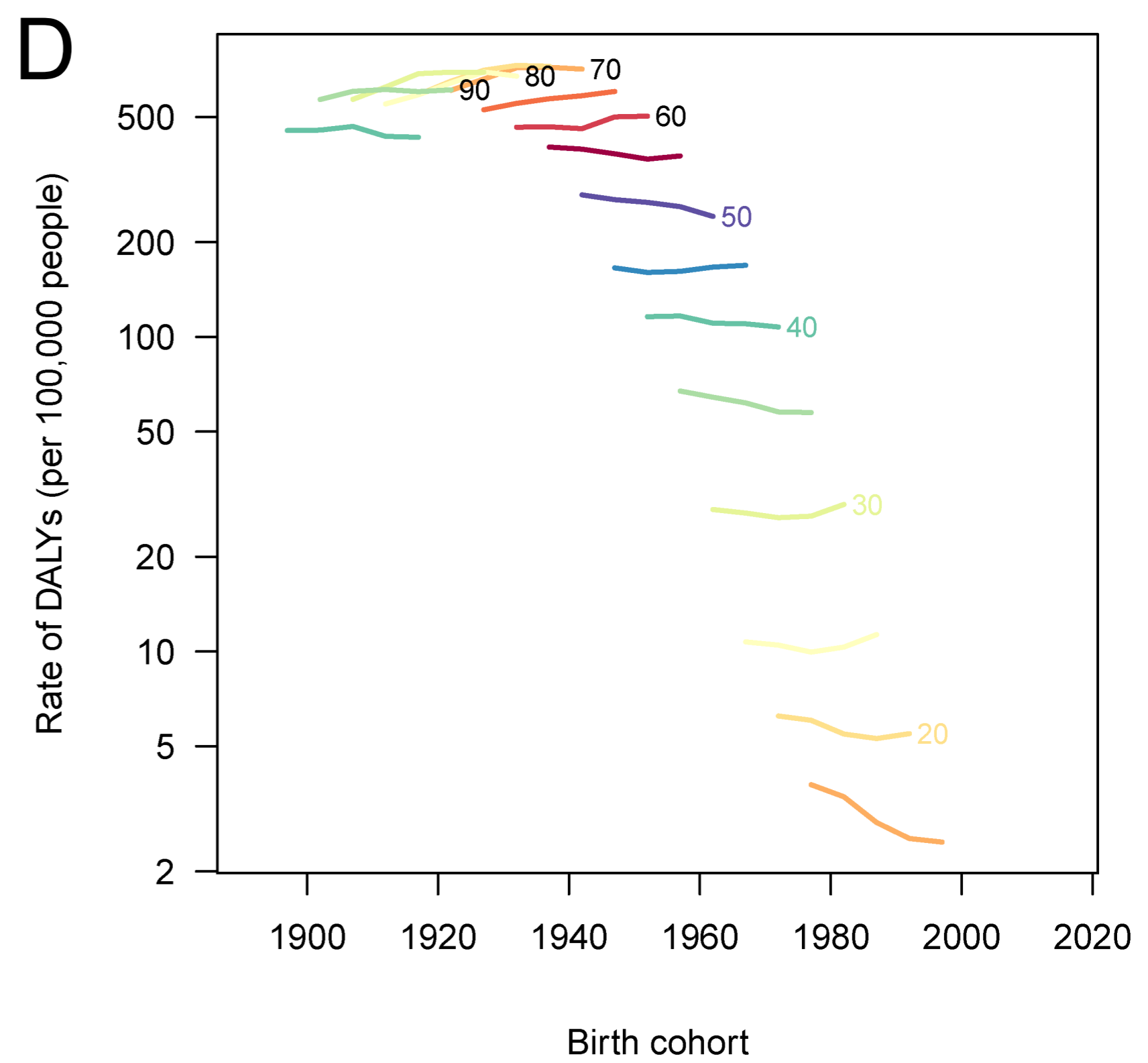

Supplement: S5 Fig — (A) Age-standardized DALY rates of pancreatic cancer according to time periods; each line connects the age-specific mortality for a 5-year period. (B) Age-standardized DALY rates of pancreatic cancer according to birth cohorts; each line connects the age-specific mortality for a 5-year birth cohort. (C) Period-specific DALY rates of pancreatic cancer according to age groups; each line connects the period-specific mortality for a 5-year age group. (D) Cohort-specific DALY rates of pancreatic cancer according to age groups; each line connects the cohort-specific mortality for a 5-year age group. DALY, disability-adjusted life year. (PDF) [file pone.0327009.s005.pdf]
