# Supplementary material for: Rising burden of pancreatic cancer in China: Trends, drivers, and future projections
Source: PLoS One. 2025 Jul 1;20(7):e0327009. doi: 10.1371/journal.pone.0327009 (PMC12212494; doi:10.1371/journal.pone.0327009)
Supplement: S1 Table — (DOCX) [file pone.0327009.s006.docx]

S1 Table. Prevalent cases, deaths and DALYs for pancreatic cancer in 2021, and percentage change in ASRs per 100000, by GBD region, from 1990 to 2021

|  | Prevalence (95% UI) | | | Deaths (95% UI) | | | DALYs (95% UI) | | |
| --- | --- | --- | --- | --- | --- | --- | --- | --- | --- |
| Location | No, in thousands (95% UI) | ASRs per 100000 (95% UI) | Percentage change in ASRs from 1990 to 2021 | No, in thousands (95% UI) | ASRs per 100000 (95% UI) | Percentage change in ASRs from 1990 to 2021 | No, in thousands (95% UI) | ASRs per 100000 (95% UI). | Percentage change in ASRs from 1990 to 2021 |
| Global | 439 (401.7, 471) | 5.1 (4.7, 5.5) | 16.5 (7.3, 25.1) | 505.8 (461.2, 543.9) | 5.9 (5.4, 6.4) | 5.2 (-3.3, 13.1) | 11317 (10464.7, 12169.3) | 130.3 (120.5, 140.1) | 0.8 (-8.5, 9.6) |
| High-income Asia Pacific | 55.5 (46.3, 61.1) | 11.2 (9.7, 12.2) | 29.3 (18.6, 38.3) | 49.6 (41.7, 54.4) | 9.6 (8.3, 10.3) | 7.1 (-1.2, 12.9) | 864.9 (760.4, 931.6) | 197.6 (178.9, 210.6) | -0.7 (-7.4, 4.7) |
| High-income North America | 62.2 (57.2, 64.9) | 9.7 (9, 10.1) | 18.4 (15.3, 21.2) | 63 (57.5, 66.2) | 9.3 (8.6, 9.8) | 4.6 (1.7, 7.3) | 1309.2 (1232, 1360) | 205.4 (194.7, 212.8) | 1.3 (-1, 3.5) |
| Western Europe | 86 (78.1, 91.8) | 9.7 (9, 10.3) | 40.6 (34.7, 46) | 91.4 (81.9, 97.8) | 9.3 (8.4, 9.8) | 8.6 (3.9, 12.7) | 1738.6 (1598.8, 1842.2) | 199.4 (185.7, 210.2) | 4.5 (0.8, 7.8) |
| Australasia | 4.9 (4.4, 5.3) | 9.3 (8.5, 9.9) | 35 (25.4, 45) | 4.3 (3.9, 4.6) | 7.7 (7, 8.2) | 4.3 (-2.9, 11.4) | 85.1 (78.2, 90.4) | 164.9 (153, 175.1) | 0.1 (-6.3, 6.6) |
| Andean Latin America | 2.3 (1.8, 2.9) | 3.9 (3, 4.9) | 20.3 (-6, 53.3) | 3.2 (2.5, 4) | 5.6 (4.3, 7) | 16.4 (-8.4, 46.2) | 74.7 (57.6, 94) | 125.2 (96.4, 157.4) | 13 (-11.4, 44.8) |
| Tropical Latin America | 11.7 (11, 12.2) | 4.5 (4.2, 4.7) | 26.9 (21.7, 31.9) | 16 (14.7, 16.7) | 6.3 (5.8, 6.6) | 21.7 (16.6, 26.6) | 382 (361.7, 398.1) | 146.5 (138.5, 152.8) | 21.4 (16.3, 26.2) |
| Central Latin America | 8.9 (8, 9.9) | 3.5 (3.1, 3.9) | 7.7 (-2.8, 19.1) | 11.9 (10.7, 13.2) | 4.8 (4.3, 5.4) | 2.1 (-7.6, 12.5) | 289.9 (260.6, 321.7) | 113.6 (102, 126.2) | 3.3 (-6.9, 14.4) |
| Southern Latin America | 5.7 (5.3, 6.1) | 6.6 (6.2, 7.1) | 0.6 (-7.6, 8.6) | 8.1 (7.5, 8.6) | 9.1 (8.4, 9.7) | -4.7 (-12.7, 3.5) | 176.4 (165.6, 187.9) | 205 (192.7, 218.3) | -5.9 (-13.5, 1.7) |
| Caribbean | 2.1 (1.9, 2.4) | 4 (3.5, 4.5) | 15.2 (0, 29.8) | 2.9 (2.6, 3.3) | 5.4 (4.8, 6.1) | 6.5 (-7.1, 20.2) | 68.1 (59.6, 77.4) | 126.5 (110.7, 144.1) | 12.5 (-2.7, 27.4) |
| Central Europe | 15.6 (14.3, 17) | 7.3 (6.7, 7.9) | 16 (6, 25.2) | 22 (20.1, 23.7) | 9.7 (8.9, 10.5) | 12.3 (2.6, 20.9) | 481.4 (442.7, 521.3) | 227.3 (208.8, 246.4) | 7.3 (-2, 16.1) |
| Eastern Europe | 22.7 (21, 24.7) | 6.6 (6.1, 7.2) | 15.2 (4.2, 27.2) | 30.1 (27.8, 32.6) | 8.5 (7.9, 9.2) | 15.6 (5.7, 26.3) | 723.8 (666.4, 786.8) | 212.4 (195.6, 230.8) | 8.4 (-1.9, 19.8) |
| Central Asia | 2.8 (2.5, 3.2) | 3.3 (2.9, 3.7) | 39.8 (18.6, 64) | 3.6 (3.2, 4) | 4.5 (4, 5.1) | 43.2 (21.3, 67.4) | 97.3 (85.2, 109.8) | 111.6 (97.9, 125.6) | 35 (14.1, 59.4) |
| North Africa and Middle East | 15.9 (13.9, 17.9) | 3.4 (3, 3.9) | 57.2 (28.4, 98.8) | 19.8 (17.4, 22.2) | 4.7 (4.1, 5.3) | 51.6 (24.4, 94.5) | 507 (444.6, 573.3) | 106.7 (93.8, 120.3) | 43.7 (16.5, 81.6) |
| South Asia | 16.3 (14.5, 18.1) | 1.1 (1, 1.2) | 41.3 (9.1, 87.9) | 21.6 (19.2, 23.8) | 1.5 (1.3, 1.7) | 41.4 (9.6, 89.1) | 561.2 (496.7, 621.8) | 36.4 (32.3, 40.2) | 35 (4, 80.1) |
| Southeast Asia | 17.9 (15.5, 20.9) | 2.6 (2.3, 3) | 54 (25.7, 85.1) | 22.4 (19.3, 26) | 3.5 (3, 4.1) | 50.2 (23, 80.2) | 598 (515.4, 697.9) | 85.9 (73.8, 99.7) | 44.9 (18, 74.5) |
| East Asia | 99.1 (79.1, 120.4) | 4.5 (3.6, 5.5) | 28.3 (-6.3, 69.3) | 123.8 (99.9, 149.4) | 5.7 (4.6, 6.9) | 19.2 (-11.9, 56.1) | 3033 (2404.6, 3684.5) | 137.2 (109.2, 166) | 12.2 (-18.5, 48.8) |
| Oceania | 0.1 (0.1, 0.2) | 1.7 (1.4, 2.2) | 25.1 (1.2, 59.7) | 0.2 (0.1, 0.2) | 2.5 (2, 3.1) | 22.6 (-0.3, 54.4) | 4.7 (3.8, 6.1) | 58.6 (47.5, 74.7) | 22.4 (-2.2, 58.2) |
| Western Sub-Saharan Africa | 2.9 (2.4, 3.4) | 1.4 (1.2, 1.6) | 91.7 (59.7, 133.6) | 3.8 (3.2, 4.4) | 2.1 (1.8, 2.4) | 91.8 (61, 134.9) | 101.3 (83.3, 119.9) | 48.3 (40.2, 56.3) | 87.1 (55.8, 128.7) |
| Eastern Sub-Saharan Africa | 2.7 (2.2, 3.4) | 1.5 (1.2, 1.9) | 23 (-1.5, 57.8) | 3.4 (2.8, 4.4) | 2.2 (1.8, 2.7) | 22.2 (-0.6, 55.5) | 94.8 (76.4, 123.3) | 51.7 (42, 66.3) | 19.2 (-5.3, 53.9) |
| Central Sub-Saharan Africa | 1 (0.7, 1.4) | 1.7 (1.2, 2.4) | 4.9 (-29.9, 54.6) | 1.2 (0.9, 1.7) | 2.5 (1.7, 3.5) | 3.8 (-30.7, 50.9) | 36.1 (24.9, 50.6) | 59.4 (41, 83) | 2.6 (-32.1, 52.8) |
| Southern Sub-Saharan Africa | 2.6 (2.3, 2.8) | 4.3 (3.8, 4.8) | 49.1 (27, 76.8) | 3.4 (3, 3.7) | 6.2 (5.5, 6.9) | 51 (27.2, 80.6) | 89.5 (78.9, 99.7) | 147.3 (129.8, 163) | 47.8 (26.1, 75.9) |

Values in parentheses indicate 95% UIs, estimated using Monte Carlo simulations. Abbreviations: DALYs, disability-adjusted life years; ASRs, age standardized rates; 95% UI, 95% uncertainty intervals.
