# Supplementary material for: Rising burden of pancreatic cancer in China: Trends, drivers, and future projections
Source: PLoS One. 2025 Jul 1;20(7):e0327009. doi: 10.1371/journal.pone.0327009 (PMC12212494; doi:10.1371/journal.pone.0327009)
Supplement: S2 Table — (DOCX) [file pone.0327009.s007.docx]

S2 Table. Joinpoint regression analysis of trends in age-standardized incidence, prevalence, mortality rates (per 100,000) by sex for pancreatic cancer in China, 1990-2021.

|  | ASIR |  |  | ASPR |  |  | ASMR |  |  |
| --- | --- | --- | --- | --- | --- | --- | --- | --- | --- |
| Gender | Period | APC (95% CI) | AAPC (95% CI) | Period | APC (95% CI) | AAPC (95% CI) | Period | APC (95% CI) | AAPC (95% CI) |
| Both | 1990-1997 | 0.35 (0.09 - 0.61) ^*^ | 0.72 (0.50 - 0.94) ^*^ | 1990-1997 | 0.25 (0.04 - 0.46) ^*^ | 0.80 (0.64 - 0.96) ^*^ | 1990-1997 | 0.33 (0.07 - 0.51) ^*^ | 0.56 (0.51 - 0.61) ^*^ |
|  | 1997-2001 | 1.45 (0.69 - 2.21) ^*^ |  | 1997-2001 | 1.44 (0.85 - 2.04) ^*^ |  | 1997-2001 | 1.49 (1.12 - 1.98) ^*^ |  |
|  | 2001-2008 | 0.44 (0.20 - 0.69) ^*^ |  | 2001-2007 | 0.40 (0.15 - 0.64) ^*^ |  | 2001-2008 | 0.19 (-0.22 - 0.30) |  |
|  | 2008-2011 | 1.65 (0.21 - 3.11) ^*^ |  | 2007-2011 | 1.56 (0.96 - 2.16) ^*^ |  | 2008-2011 | 1.29 (0.74 - 1.60) ^*^ |  |
|  | 2011-2015 | -0.71 (-1.62 - 0.20) |  | 2011-2015 | -0.33 (-1.07 - 0.41) |  | 2011-2015 | -0.96 (-1.57 - -0.59) ^*^ |  |
|  | 2015-2021 | 1.48 (1.01 - 1.95) ^*^ |  | 2015-2021 | 1.69 (1.31 - 2.07) ^*^ |  | 2015-2021 | 1.32 (0.98 - 1.76) ^*^ |  |
| Female | 1990-1997 | 0.51 (0.26 - 0.77) ^*^ | 0.48 (0.22 - 0.73) ^*^ | 1990-1997 | 0.44 (0.23 - 0.65) ^*^ | 0.52 (0.31 - 0.73) ^*^ | 1990-1997 | 0.49 (-0.38 - 0.80) | 0.33 (0.26 - 0.39) ^*^ |
|  | 1997-2000 | 1.39 (-0.04 - 2.84) |  | 1997-2000 | 1.37 (0.21 - 2.55) ^*^ |  | 1997-2000 | 1.36 (-0.52 - 1.67) |  |
|  | 2000-2008 | -0.18 (-0.36 - 0.01) |  | 2000-2008 | -0.22 (-0.37 - -0.07) ^*^ |  | 2000-2011 | -0.28 (-1.62 - -0.04) ^*^ |  |
|  | 2008-2011 | 0.45 (-0.95 - 1.86) |  | 2008-2011 | 0.58 (-0.56 - 1.74) |  | 2011-2014 | -1.83 (-2.20 - 1.30) |  |
|  | 2011-2014 | -1.78 (-3.47 - -0.06) ^*^ |  | 2011-2014 | -1.51 (-2.88 - -0.12) ^*^ |  | 2014-2021 | 1.65 (1.22 - 2.16) ^*^ |  |
|  | 2014-2021 | 1.81 (1.48 - 2.14) ^*^ |  | 2014-2021 | 1.94 (1.66 - 2.21) ^*^ |  |  |  |  |
| Male | 1990-1997 | 0.25 (-0.02 - 0.51) | 0.89 (0.68 - 1.10) ^*^ | 1990-1997 | 0.17 (-0.08 - 0.41) | 1.00 (0.81 - 1.20) ^*^ | 1990-1997 | 0.24 (-0.08 - 0.49) | 0.71 (0.65 - 0.77) ^*^ |
|  | 1997-2004 | 1.57 (1.32 - 1.83) ^*^ |  | 1997-2004 | 1.52 (1.28 - 1.76) ^*^ |  | 1997-2004 | 1.52 (1.36 - 1.86) ^*^ |  |
|  | 2004-2007 | 0.36 (-0.90 - 1.64) |  | 2004-2007 | 0.51 (-0.66 - 1.69) |  | 2004-2007 | -0.07 (-0.41 - 0.48) |  |
|  | 2007-2011 | 2.13 (1.36 - 2.90) ^*^ |  | 2007-2011 | 2.33 (1.63 - 3.03) ^*^ |  | 2007-2011 | 1.84 (1.47 - 2.42) ^*^ |  |
|  | 2011-2016 | -0.19 (-0.77 - 0.41) |  | 2011-2016 | 0.14 (-0.41 - 0.68) |  | 2011-2015 | -0.59 (-1.19 - -0.06) ^*^ |  |
|  | 2016-2021 | 1.26 (0.61 - 1.92) ^*^ |  | 2016-2021 | 1.57 (0.98 - 2.18) ^*^ |  | 2015-2021 | 0.85 (0.48 - 1.72) ^*^ |  |

Values in parentheses indicate 95% UIs, estimated using Monte Carlo simulations. Abbreviations: ASIR, age-standardized incidence rates; ASMR, age-standardized mortality rates; ASPR, age-standardized prevalence rates; AAPC, average annual percent change presented for full period; APC, annual percent change; CI, confidence interval. ^*^, *p* <0.05.
