# Supplementary material for: Rising burden of pancreatic cancer in China: Trends, drivers, and future projections
Source: PLoS One. 2025 Jul 1;20(7):e0327009. doi: 10.1371/journal.pone.0327009 (PMC12212494; doi:10.1371/journal.pone.0327009)
Supplement: S3 Table — (DOCX) [file pone.0327009.s008.docx]

S3 Table. Joinpoint regression analysis of trends in age-standardized DALYs, YLDs, and YLLs rates (per 100,000) by sex for pancreatic cancer in China, 1990-2021.

|  | DALYs |  |  | YLDs |  |  | YLLs |  |  |
| --- | --- | --- | --- | --- | --- | --- | --- | --- | --- |
| Gender | Period | APC (95% CI) | AAPC (95% CI) | Period | APC (95% CI) | AAPC (95% CI) | Period | APC (95% CI) | AAPC (95% CI) |
| Both | 1990-1997 | -0.05 (-0.25 - 0.14) | 0.36 (0.18 - 0.54) ^*^ | 1990-1997 | 0.24 (0.06 - 0.42) ^*^ | 0.66 (0.47 - 0.86) ^*^ | 1990-1997 | -0.06 (-0.27 - 0.08) | 0.36 (0.31 - 0.39) ^*^ |
|  | 1997-2000 | 1.20 (0.07 - 2.33) ^*^ |  | 1997-2000 | 1.46 (0.18 - 2.76) ^*^ |  | 1997-2000 | 1.19 (0.75 - 1.40) ^*^ |  |
|  | 2000-2008 | 0.01 (-0.13 - 0.15) |  | 2000-2008 | 0.43 (0.26 - 0.60) ^*^ |  | 2000-2008 | 0.00 (-0.16 - 0.07) |  |
|  | 2008-2011 | 1.25 (0.17 - 2.33) ^*^ |  | 2008-2011 | 1.62 (0.34 - 2.92) ^*^ |  | 2008-2011 | 1.24 (0.78 - 1.47) ^*^ |  |
|  | 2011-2015 | -0.85 (-1.54 - -0.16) ^*^ |  | 2011-2015 | -0.59 (-1.24 - 0.06) |  | 2011-2015 | -0.85 (-1.32 - -0.59) ^*^ |  |
|  | 2015-2021 | 1.27 (0.91 - 1.63) ^*^ |  | 2015-2021 | 1.44 (1.20 - 1.69) ^*^ |  | 2015-2021 | 1.27 (1.00 - 1.64) ^*^ |  |
| Female | 1990-1997 | 0.13 (-0.06 - 0.32) | 0.07 (-0.12 - 0.25) | 1990-1997 | 0.44 (0.24 - 0.63) ^*^ | 0.43 (0.22 - 0.65) ^*^ | 1990-1997 | 0.12 (-0.30 - 0.28) | 0.06 (0.02 - 0.10) ^*^ |
|  | 1997-2000 | 0.91 (-0.13 - 1.97) |  | 1997-2000 | 1.22 (-0.16 - 2.62) |  | 1997-2000 | 0.91 (0.43 - 1.15) ^*^ |  |
|  | 2000-2008 | -0.79 (-0.93 - -0.66) ^*^ |  | 2000-2007 | -0.37 (-0.61 - -0.14) ^*^ |  | 2000-2008 | -0.80 (-1.05 - -0.72) ^*^ |  |
|  | 2008-2011 | -0.03 (-1.04 - 0.98) |  | 2007-2011 | 0.39 (-0.31 - 1.09) |  | 2008-2011 | -0.04 (-0.43 - 0.17) |  |
|  | 2011-2014 | -1.89 (-3.14 - -0.63) ^*^ |  | 2011-2014 | -1.54 (-2.99 - -0.07) ^*^ |  | 2011-2014 | -1.89 (-2.16 - -1.44) ^*^ |  |
|  | 2014-2021 | 1.54 (1.29 - 1.79) ^*^ |  | 2014-2021 | 1.79 (1.56 - 2.01) ^*^ |  | 2014-2021 | 1.53 (1.33 - 1.72) ^*^ |  |
| Male | 1990-1996 | -0.25 (-0.54 - 0.05) | 0.55 (0.36 - 0.73) ^*^ | 1990-1997 | 0.14 (-0.06 - 0.33) | 0.83 (0.65 - 1.02) ^*^ | 1990-1996 | -0.25 (-0.68 - 0.04) | 0.55 (0.48 - 0.60) ^*^ |
|  | 1996-2004 | 0.95 (0.77 - 1.12) ^*^ |  | 1997-2004 | 1.40 (1.17 - 1.63) ^*^ |  | 1996-2004 | 0.94 (0.83 - 1.29) ^*^ |  |
|  | 2004-2007 | -0.03 (-1.14 - 1.09) |  | 2004-2007 | 0.39 (-1.01 - 1.81) |  | 2004-2007 | -0.04 (-0.33 - 0.43) |  |
|  | 2007-2011 | 1.79 (1.13 - 2.45) ^*^ |  | 2007-2011 | 2.11 (1.40 - 2.81) ^*^ |  | 2007-2011 | 1.79 (1.42 - 2.31) ^*^ |  |
|  | 2011-2016 | -0.36 (-0.88 - 0.15) |  | 2011-2016 | -0.14 (-0.61 - 0.33) |  | 2011-2016 | -0.37 (-0.96 - -0.01) ^*^ |  |
|  | 2016-2021 | 1.15 (0.58 - 1.72) ^*^ |  | 2016-2021 | 1.25 (0.88 - 1.63) ^*^ |  | 2016-2021 | 1.15 (0.67 - 2.25) ^*^ |  |

Values in parentheses indicate 95% UIs, estimated using Monte Carlo simulations. Abbreviations: DALYs, disability-adjusted life years; YLDs, years lived with disability; YLLs, years of life lost; AAPC, average annual percent change presented for full period; APC, annual percent change; CI, confidence interval. ^*^, *p* <0.05
